# Genomic analyses of two novel biofilm-degrading methicillin-resistant *Staphylococcus aureus* phages

**DOI:** 10.1186/s12866-019-1484-9

**Published:** 2019-05-28

**Authors:** Khulood Hamid Dakheel, Raha Abdul Rahim, Vasantha Kumari Neela, Jameel R. Al-Obaidi, Tan Geok Hun, Mohd Noor Mat Isa, Khatijah Yusoff

**Affiliations:** 1 Department of Microbiology, Faculty of Biotechnology and Biomolecular Sciences, Universiti Putra Malaysia, 43400 Serdang, Selangor Darul Ehsan Malaysia; 2 Department of Cell and Molecular Biology, Faculty of Biotechnology and Biomolecular Sciences, Universiti Putra Malaysia, 43400 Serdang, Selangor Darul Ehsan Malaysia; 3 Institute of Bioscience, Universiti Putra Malaysia, 43400 Serdang, Selangor Darul Ehsan Malaysia; 4 Department of Medical Microbiology and Parasitology, Faculty of Medicine and Health Sciences, Universiti Putra Malaysia, 43400 Serdang, Selangor Darul Ehsan Malaysia; 5 Agro-biotechnology Institute Malaysia (ABI), c/o MARDI Headquarters, 43400 Serdang, Selangor Malaysia; 6 Department of Agriculture Technology, Faculty of Agriculture, Universiti Putra Malaysia, 43400 Serdang, Selangor Malaysia; 7 Malaysia Genome Institute (MGI), Jalan Bangi, 43000 Kajang, Selangor Malaysia; 8 Department of Biology, College of Science, Mustansiriyah University, Palestine Street, PO Box 14022, Baghdad, Iraq

**Keywords:** MRSA biofilm, Bacteriophage, Virus, Confocal laser scanning microscopy (CLSM), Microtiter plates

## Abstract

**Background:**

Methicillin-resistant *Staphylococcus aureus* (MRSA) biofilm producers represent an important etiological agent of many chronic human infections. Antibiotics and host immune responses are largely ineffective against bacteria within biofilms. Alternative actions and novel antimicrobials should be considered. In this context, the use of phages to destroy MRSA biofilms presents an innovative alternative mechanism.

**Results:**

Twenty-five MRSA biofilm producers were used as substrates to isolate MRSA-specific phages. Despite the difficulties in obtaining an isolate of this phage, two phages (UPMK_1 and UPMK_2) were isolated. Both phages varied in their ability to produce halos around their plaques, host infectivity, one-step growth curves, and electron microscopy features. Furthermore, both phages demonstrated antagonistic infectivity on planktonic cultures. This was validated in an in vitro static biofilm assay (in microtiter-plates), followed by the visualization of the biofilm architecture in situ via confocal laser scanning microscopy before and after phage infection, and further supported by phages genome analysis. The UPMK_1 genome comprised 152,788 bp coding for 155 putative open reading frames (ORFs), and its genome characteristics were between the *Myoviridae* and *Siphoviridae* family, though the morphological features confined it more to the *Siphoviridae* family. The UPMK_2 has 40,955 bp with 62 putative ORFs; morphologically, it presented the features of the *Podoviridae* though its genome did not show similarity with any of the *S. aureus* in the *Podoviridae* family. Both phages possess lytic enzymes that were associated with a high ability to degrade biofilms as shown in the microtiter plate and CLSM analyses.

**Conclusions:**

The present work addressed the possibility of using phages as potential biocontrol agents for biofilm-producing MRSA.

**Electronic supplementary material:**

The online version of this article (10.1186/s12866-019-1484-9) contains supplementary material, which is available to authorized users.

## Background

The susceptibility of both planktonic bacteria and biofilm-associated bacteria varies; the latter being more resistant to treatments with standard chemical antibacterial agents due to the different resistance mechanisms in the biofilm community. In the light of emerging antibiotic resistance among biofilm producing bacteria and antibiotic-induced biofilm production, the development of selective antibacterial agents with less toxicity towards the treatment of biofilm-induced infections has become imperative [1, 2]. Therefore, the phage-based anti-biofilm strategy would be an attractive solution towards addressing the biofilm menace. Bacteriophages, like all viruses, are obligate intracellular parasites that need a host to multiply. They require actively growing host cells to multiply and reproduce [3]. They are abundant in the water environment [4–6].

Sewage samples are always ideal for the isolation of phages for enteric bacteria as they contain diverse enteric bacterial hosts. Additional promising sampling locations include surface water such as lakes, rivers, and canals since these frequently receive fecal material from animals.

Lytic bacteriophages isolated and characterized from several MSRA strains play crucial roles in the investigation of the potential use of phages and their products as therapeutic agents against infections caused by biofilm-producing MRSA. The difficulties associated with the isolation of bacteriophages have been well documented [7–13]. Sample enrichment is the best approach for the isolation of promising phages. It is usually performed in situations where there are small numbers of the targeted phages, allowing their propagation when the samples are incubated with some of the relevant clinical isolates [3, 14].

Bacteriophages have been tested as anti-infectives in human and animals [15, 16]. Phage encoded lytic proteins have also been used to inhibit pathogenic bacteria [17–19]. In most cases, the evaluation of the phage lytic activity is performed against planktonic cultures only, which to some extent, is limited by the approach used. Generally, bacterial cells tend to be in biofilm communities, where they switch their forms in a protective strategy against hostile environments [20]. The capability of single phages, phage cocktail and phage recombinant proteins to remove *S. aureus* biofilms on food matrices and on clinical models is very complex process [21–26]. Some phages possess specific hydrolytic enzymes which enhance their invasion and dispersion processes through biofilms towards the infection of other bacteria [27]. Doolittle et al. [28] showed that progeny phage will propagate radially through a biofilm. Theoretically, the dense adjacent cells within the biofilm would be killed and the biofilm matrix degraded by a single phage dose. The difficulties that the phages may encounter in eliminating the bacteria in the matrix of biofilms were reported by Cerca et al. [29], who showed that phage K was able to reduce a planktonic culture of *Staphylococcus epidermidis* within 2 h, while requiring 24 h to reduce a biofilm biomass after infection. This was due to the presence of the protective and cell aggregating *S. epidermidis* extracellular polysaccharide and the poly-N-acetylglucosamine (PNAG) expressed by the cells in the biofilm as well as to the low metabolic activity of biofilm cells. Other studies, though, have shown efficient phage infection within mature (seven-old-day) biofilms [30].

Therefore, to more accurately reflect the assessment of the lytic effect of phages against bacteria within biofilms, it is recommended to study this effect in biofilm models. Several in vitro models for biofilm formation have been developed over the years. In this study, bacteriophages from the environment which can degrade MRSA biofilms were isolated, characterized, and evaluated for their ability to degrade biofilms.

## Results

### Bacteriophage isolation and phage morphology

**Fig. 1:**
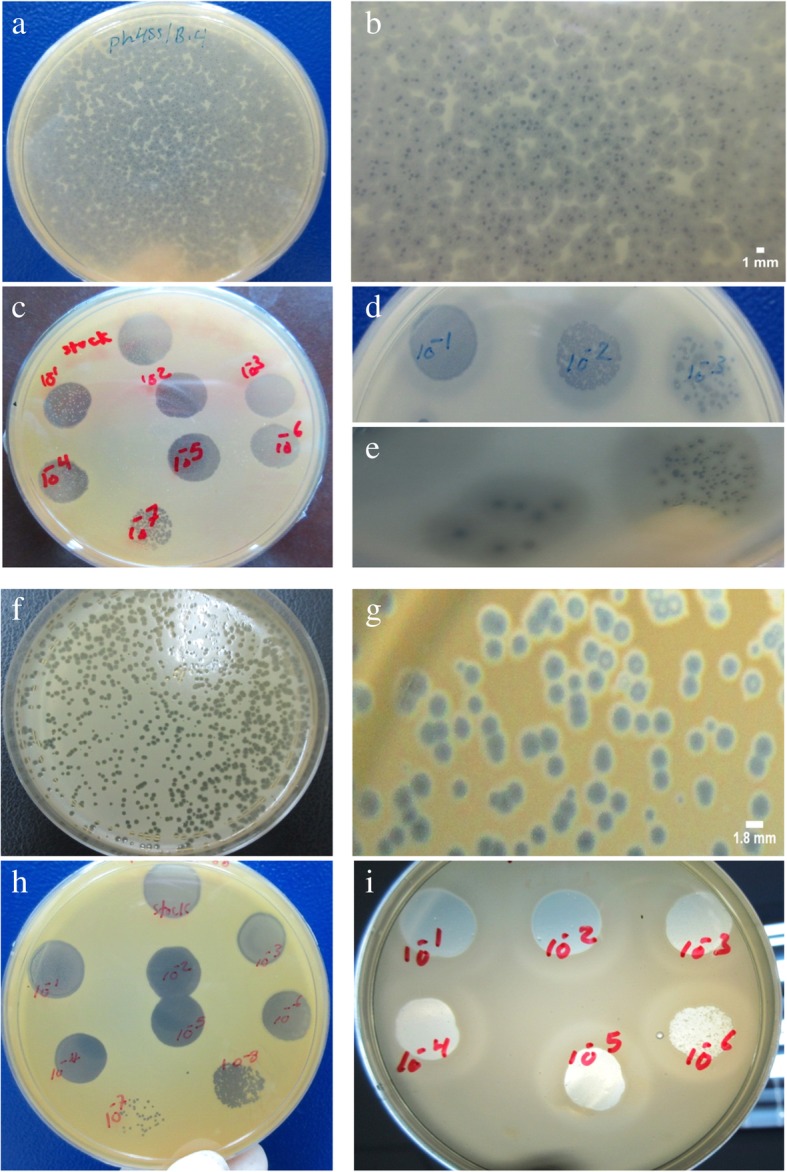
Examples of MRSA isolated phage plaques. **a**, **b**, **c**, **d** and **e** represent phage UPMK_1 infected bacterial lawn host MRSA t127/4, while **f**, **g**, **h** and **i** represent phage UPMK_2 infected bacterial lawn host MRSA t223/20. Using 0.6% agar overlay method plaques morphology of UPMK_1 (B and E) and UPMK_2 (G) appeared as lysis center surrounded by turbid halos. The phage lysate in plates C, D, H, I was applied in 10-fold serial dilutions to lawn of the host bacteria by spotting 10 μl from the phage dilution. The turbid halo around the lysis zone appeared clearer and its size increased as shown in D and I after longer incubation

Two phages (UPMK_1 and UPMK_2) were successfully isolated from UPM park lakes and sewage water, respectively. The isolated phages (UPMK_1 and UPMK_2) produced confluent clear lyses on MRSAt127/4 and MRSAt223/20 respectively. Both MRSAt127/4 and MRSAt223/20 are PIA-dependent biofilms producers. The plaque morphology of both phages showed them to have circular and regular borders, and they were generally small in diameter (0.3 mm to 1.5 mm for UPMK_1 and 2 mm for UPMK_2). Both phages had halo zones surrounding the plaques, as shown in Fig. 1 b, e and g. The halo size was observed to increase with the time of incubation when kept at room temperature for 1 week, as shown in Fig. 1 d and i.

### Phage lytic assessment and sensitivity screening

**Fig. 2:**
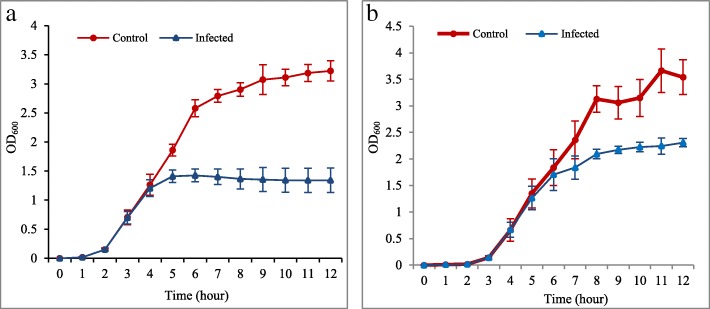
Determination of phage lytic efficiency at an optical density of 600 nm. The quantification of the culture cell density before and after infection with the bacteriophage as at MOI = 1; (**a**) = UPMK_1 with its host MRSAt127/4, while (**b**) = UPMK_2 with its host MRSAt223/20. Error bars represent mean values ± standard deviation of three independent experiments

The phage lytic assay based on the bacteriolytic activity of phage UPMK_1 and UPMK_2 on MRSA t127/4 and MRSA t223/20 respectively, showed a decrease in the bacterial optical density after treatment compared to the untreated samples, as shown in Fig. 2a and b.

**Fig. 3:**
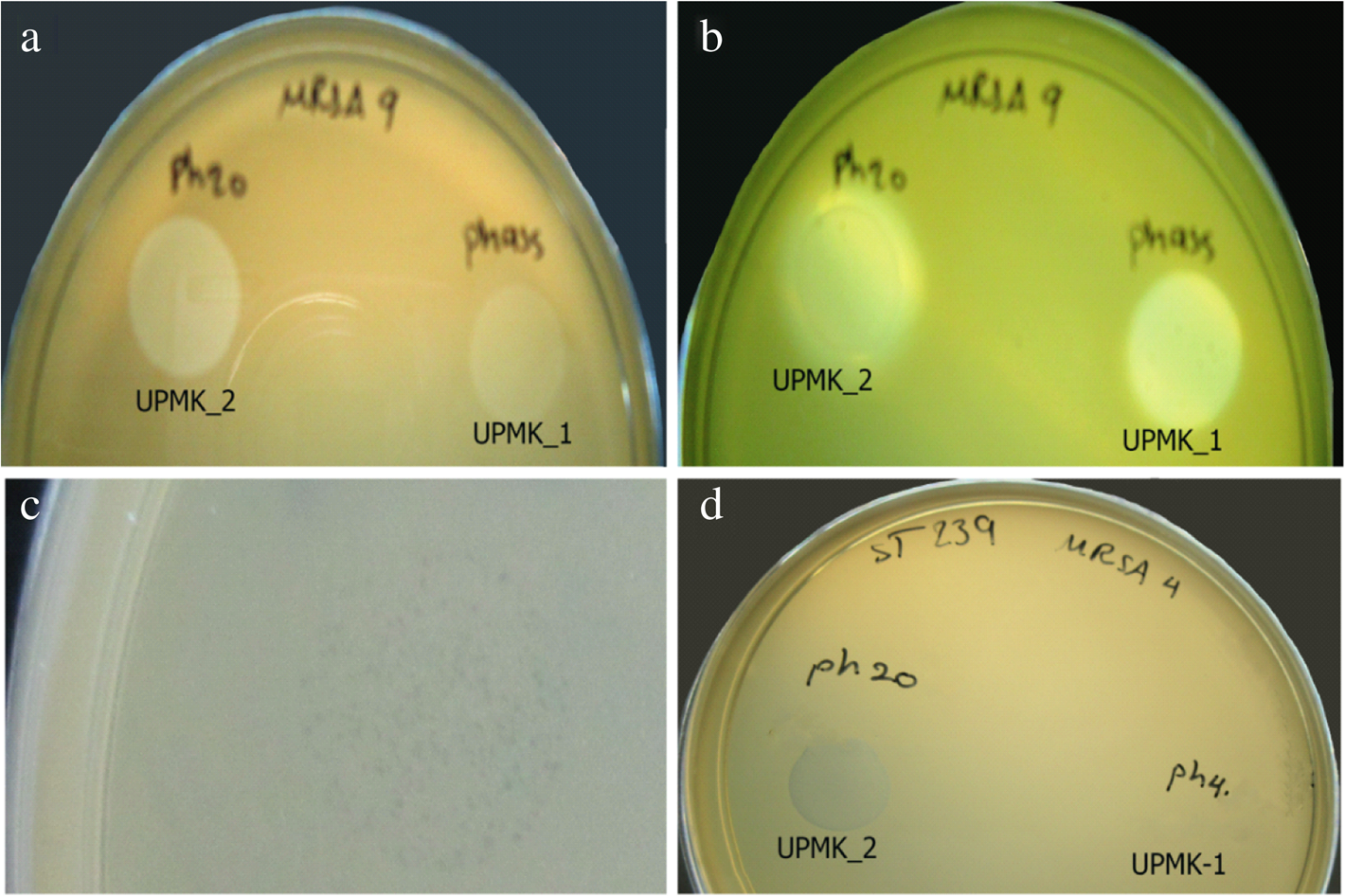
Spot test method; ph 4 represents UPMK_1 and ph 20 represents UPMK_2 (**a**) MRSAt2249/9 after 24 h of adding 10 μL of UPMK_1 and UPMK_2; halo was observed around UPMK_2 spot when incubated at room temperature for a week (**b**); (**c**) Small plaques formed by other MRSA isolates during EOP assay; (**d**) Clear lysis spot as a result of adding 10 μL of UPMK_2 on MRSA ST239 compared to the addition of UPMK_1

As shown in Table 1, both UPMK_1 and UPMK_2 were active on the 25 MRSA isolates. The majority of the MRSA isolates were considered to be closely related to *spa* type t127 and their biofilm matrix is a complex of DNA-proteins without or with exopolysaccharides. The MRSAt127/11, t790, and t223 were resistant to UPMK_1 but susceptible to UPMK_2; while MRSAt127/6 and t127/7 were resistant to both UPMK_1 and UPMK_2. Interestingly, MRSAt2246/9, t127/13, t127/17, and t127/21 had halos around the lysed area produced from the spot test with phage UPMK_2 when incubated at room temperature for a week (Fig. 3a and b). No such activity has been observed for other isolates with UPMK_2 nor with UPMK_1. Based on the criteria of Viazis et al. [31] on the classification of the efficiency of plating (EOP) values, phage UPMK_2 showed a high productivity on MRSAt127/3, t127/4, and t127/18 compared to UPMK_1. In addition, UPMK_1 exhibited slightly higher EOP in some PIA-independent biofilm producers than in PIA-dependent biofilm producers. It was observed that all the tested isolates produced smaller plaques (Fig. 3c), compared to plaques produced in the original bacterial host. From the phage lytic ability on other MRSA strains, only UPMK_2 showed a positive lytic activity against 25 MRSA strains related to ST239; and despite the unobserved individual plaques, clear spots were, however, detected (Fig. 3d). The growth of most of the MRSA (ST239) strains on the double layer plate assay was inhibited by UPMK_2.

**Table 1:** The host range and EOP for UPMK_1 and UPMK_2

Indicator host	UPMK_1/MRSAt127/4^a^			UPMK_2/MRSAt223/20^a^		
	Spot test	Titer PFU/mL	EOP	Spot test	Titer PFU/mL	EOP
MRSA t127/1	I	1 × 10^4^	1 × 10^−6^	I	1 × 10^5^	1 × 10^−5^
MRSA t127/2	I	1 × 10^5^	1 × 10^−5^	S	1 × 10^6^	1 × 10^−4^
MRSA t127/3	S	1 × 10^9^	0.1	S	1 × 10^10^	1
MRSA t127/4	Phage production host			S	1 × 10^10^	1
MRSA t127/5	S	1 × 10^8^	1 × 10^−2^	S	1 × 10^7^	1 × 10^−3^
MRSA t127/6	R	–	–	R	–	–
MRSA t127/7	R	–	–	R	–	–
MRSA t127/8	I	1 × 10^4^	1 × 10^−6^	S	1 × 10^6^	1 × 10^−4^
MRSA t2246/9	I	1 × 10^4^	1 × 10^−6^	I	1 × 10^5^	1 × 10^−5^
MRSA t127/10	I	1 × 10^4^	1 × 10^−6^	S	1 × 10^6^	1 × 10^−4^
MRSA t127/11	R	–	–	I	1 × 10^5^	1 × 10^−5^
MRSA t127/12	S	1 × 10^6^	1 × 10^−4^	S	1 × 10^7^	1 × 10^−3^
MRSA t127/13	I	1 × 10^4^	1 × 10^−6^	S	1 × 10^6^	1 × 10^−4^
MRSA t127/14	S	1 × 10^6^	1 × 10^−4^	S	1 × 10^7^	1 × 10^−3^
MRSA t127/15	I	1 × 10^4^	1 × 10^−6^	S	1 × 10^7^	1 × 10^−3^
MRSA t127/16	S	6 × 10^7^	1 × 10^−3^	S	1 × 10^7^	1 × 10^−3^
MRSA t127/17	S	1 × 10^6^	1 × 10^−4^	S	1 × 10^6^	1 × 10^−4^
MRSA t127/18	S	1 × 10^7^	1 × 10^−3^	S	1 × 10^10^	1
MRSA t790/19	R	–	–	I	1 × 10^2^	1 × 10^−8^
MRSA t223/20	R	–	–	Phage production host		
MRSA t127/21	I	1 × 10^4^	1 × 10^−6^	S	1 × 10^7^	1 × 10^−3^
MRSA t127/22	I	1 × 10^4^	1 × 10^−6^	S	1 × 10^6^	1 × 10^−4^
MRSA t127/23	S	1 × 10^6^	1 × 10^− 4^	S	1 × 10^7^	1 × 10^−3^
MRSAt127/24	I	1 × 10^4^	1 × 10^−6^	I	1 × 10^5^	1 × 10^−5^
MRSAt127/25	I	1 × 10^4^	1 × 10^−6^	S	1 × 10^6^	1 × 10^−4^

^a^ Phage production host used; MRSA t127 represents *spa* type 127, MRSA t223 represents *spa* type 223

A titer of 1 × 10^10^ PFU/mL for UPMK_1 and UPMK_2 was used in both spot test and EOP

The host range and susceptibility of the bacterial isolates were the clear spot (susceptible; S), turbid spot (intermediate; I), and no plaque formation or killing effect detected (resistant; R)

The considered EOP values were EOP > 0.5 for high production, 0.1 < EOP < 0.5 for medium production, 0.001 < EOP < 0.1 for low production, and EOP < 0.001 for very low production

The data were presented as the mean of triplicate independent measurements

### Bacteriophage morphology

**Fig. 4:**
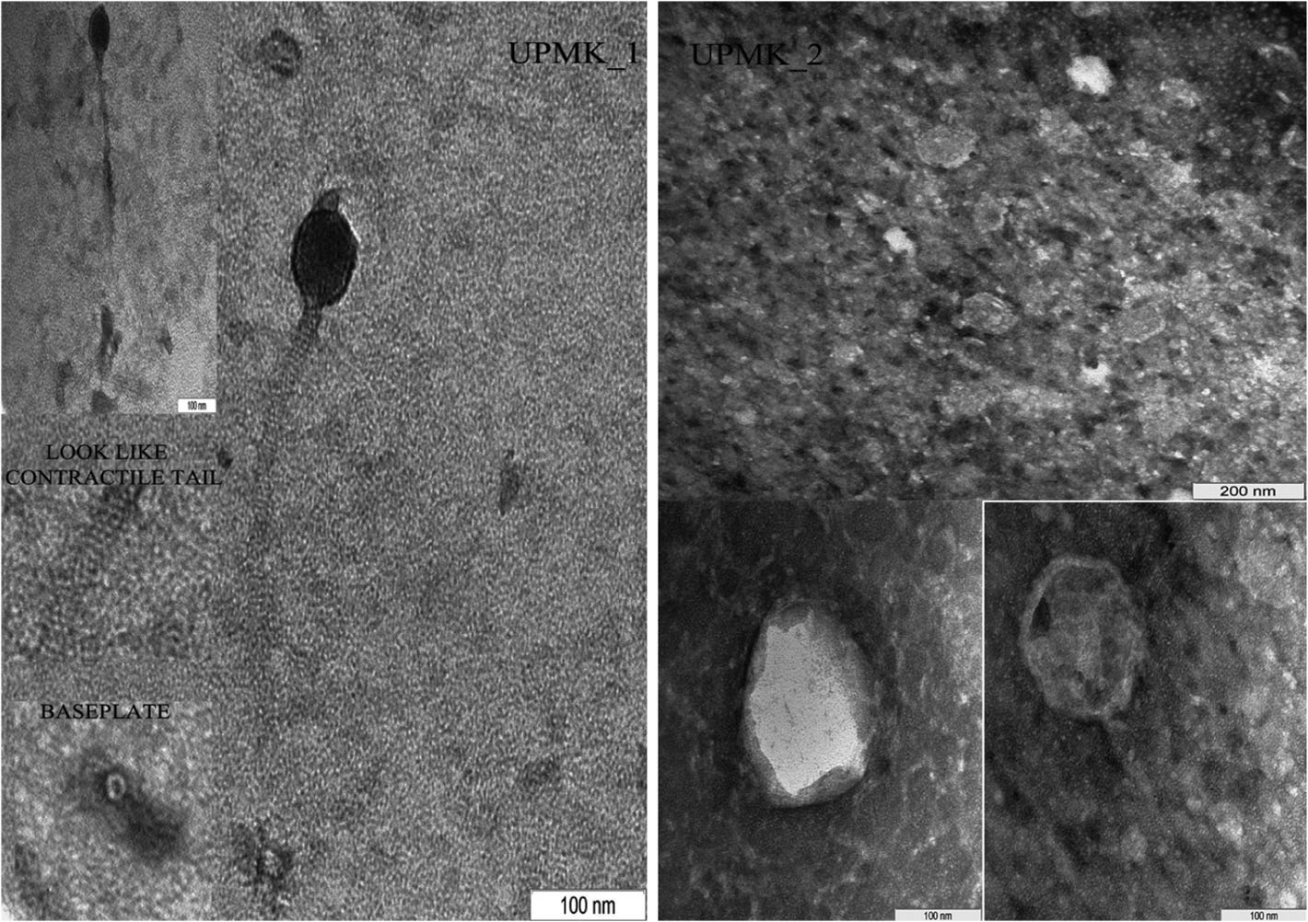
Electron micrographs of UPMK_1 (left) and UPMK_2 (right) infected MRSA-negatively stained with 1% uranyl acetate

The isolated phages (UPMK_1 and UPMK_2) were further characterized based on their morphological features. The phages were examined under a transmission electron microscopy (TEM) and shown in Fig. 4. For their detailed classification, their head diameter (width perpendicular to the tail), tail diameter, and tail length were determined. The results revealed that both phages had different morphologies, having icosahedral heads of 55.5 ± 1.5 nm and 110.5 ± 59.8 nm in diameter, as well as tail lengths of 335.9 ± 30.5 nm and 28.3 ± 15.5 nm for UPMK_1 and UPMK_2, respectively. Phage UPMK_2 can therefore, be classified as *Podoviridae* family. On the other hand, UPMK_1 had a tail diameter of 12.8 ± 1.5 nm and based on the ICTV classification [32], members of the *Myoviridae* family have a tail diameter of 16–20 nm, while the members of the *Siphoviridae* family have a tail diameter of 5–10 nm. Ackermann [33] had previously classified bacteriophages with a tail diameter of less than 16 nm which belong to *Siphoviridae* family. Despite this ruling, the Twort-like phage vB_BceM_Bc431v3 has a tail width of 12 ± 4 nm but belong to the *Myoviridae* family, leading to a blurred boundary between the phage families [34]. Therefore, the morphology and genome structure of the isolated phages in this study should guide their classification and based on the ICTV classification [32], both phages belong to the order *Caudovirales.*

### Phage growth characteristics

The adsorption affinity and life cycle of phage UPMK_1 and UPMK_2 were assessed when growing in the MRSA host cell at 37 °C in the presence of CaCl_2_. As shown in Table 2, 70% of UPMK_1 adsorbed to the host cell after 15 min while 85% of UPMK_2 adsorbed to the host cell within 15 min. Furthermore, the one-step growth analysis revealed that while phage UPMK_1 had a relatively long latency period, and a small burst size, phage UPMK_2 had a shorter latency period and a larger burst size.

**Table 2:** Percentage of free UPMK_1 and UPMK_2 after the infection of actively growing MRSA t127/4 and MRSAt223/20 at MOI =0.01

	UPMK_1		UPMK_2	
Time(min)	Free phage ±St.D	%	Free phage ±St.D	%
0	(9 ± 1) E+ 11	100	(8.66 ± 0.57) E+ 16	100.00
3	(6.33 ± 1.52) E+ 11	70.37	(6.66 ± 1.15) E+ 16	76.92
6	(4.33 ± 1.15) E+ 11	48.14	(5.33 ± 1.52)E+ 16	61.53
9	(3.66 ± 0.57) E+ 11	40.74	(4.66 ± 1.15) E+ 16	53.84
12	(3.33 ± 1.15) E+ 11	37.03	(2.66 ± 0.57) E+ 16	30.76
15	(2.66 ± 0.57) E+ 11	29.62	(1.33 ± 0.57) E+ 16	15.38

The data is presented as the mean of three independent experiments

**Fig. 5:**
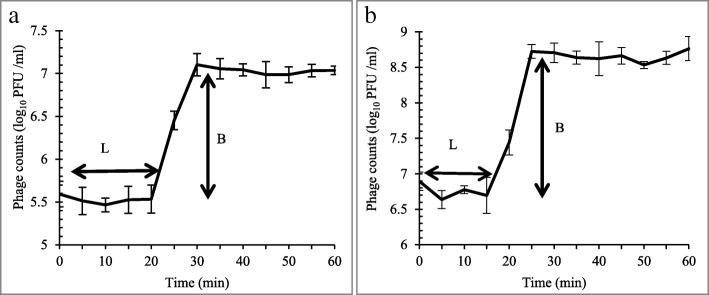
One-step phage growth was performed for UPMK_1/ MRSA t127/4 (**a**) and UPMK_2/ MRSA t223/20 (**b**) at 37 °C. The phage growth parameters are indicated in the figure and correspond to: L-latent period and B-burst size. Data points represent the mean of three independent experiments while the error bars are the mean ± standard deviations of the data sets

As shown in Fig. 5, the latent periods of UPMK_1 and UPMK_2 were 20 min and 15 min, respectively. UPMK_1 and UPMK_2 had a burst size of 32 PFU and 67 PFU per infected cell respectively, The burst size values for both phages, however, did not fit the category (more than 100 PFU/cell) for a highly effective lytic phage and this property is necessary for phage therapy [35, 36].

### Genomic analysis and characterisation of UPMK_1 and UPMK_2

**Fig. 6:**
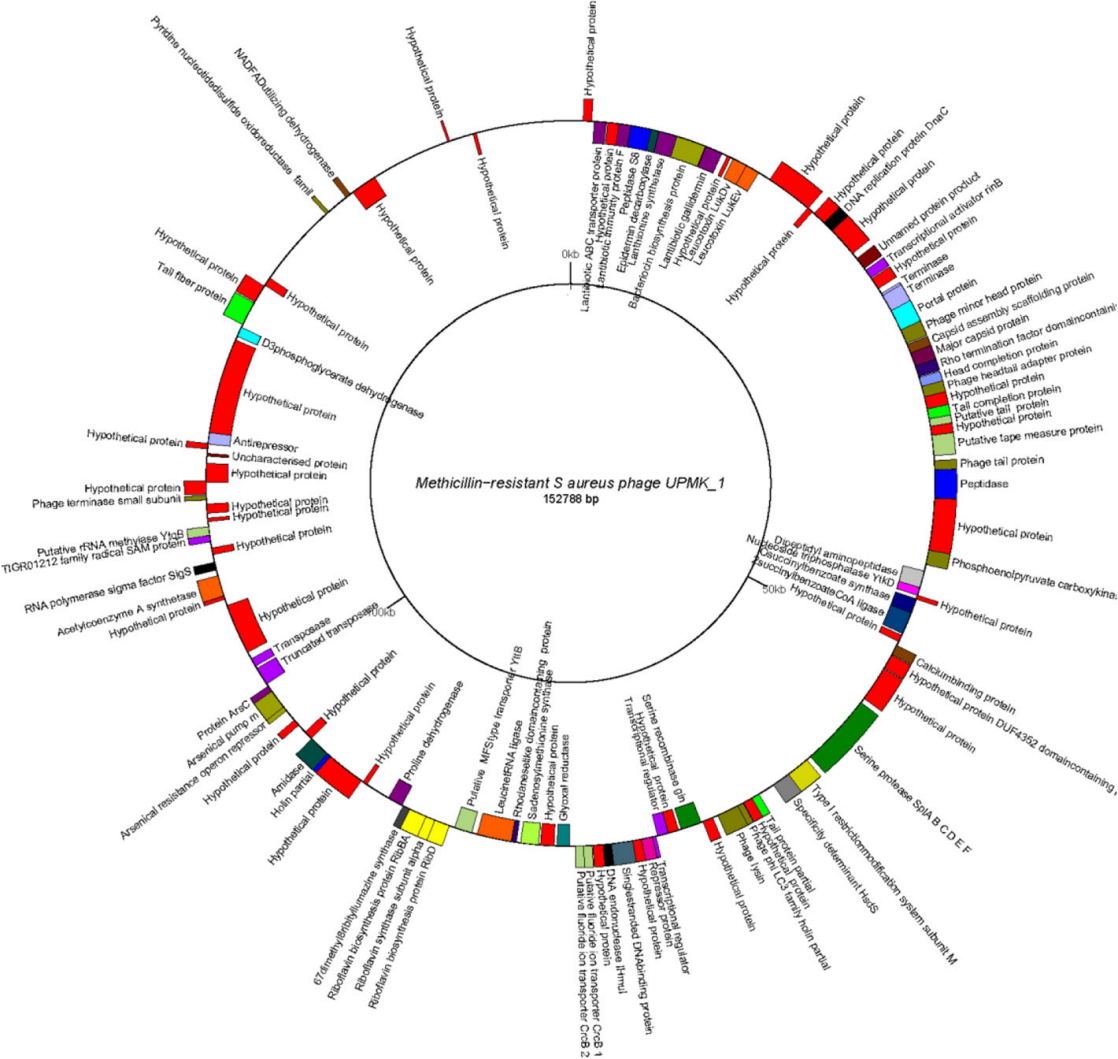
Genome map of UPMK_1 generated using GenomeVx available at http://wolfe.gen.tcd.ie/GenomeVx. Genes are shown as colored arcs and are labeled with the annotation proteins. Genes on outside are forward strand genes and genes on the inside are reverse strand genes

**Fig. 7:**
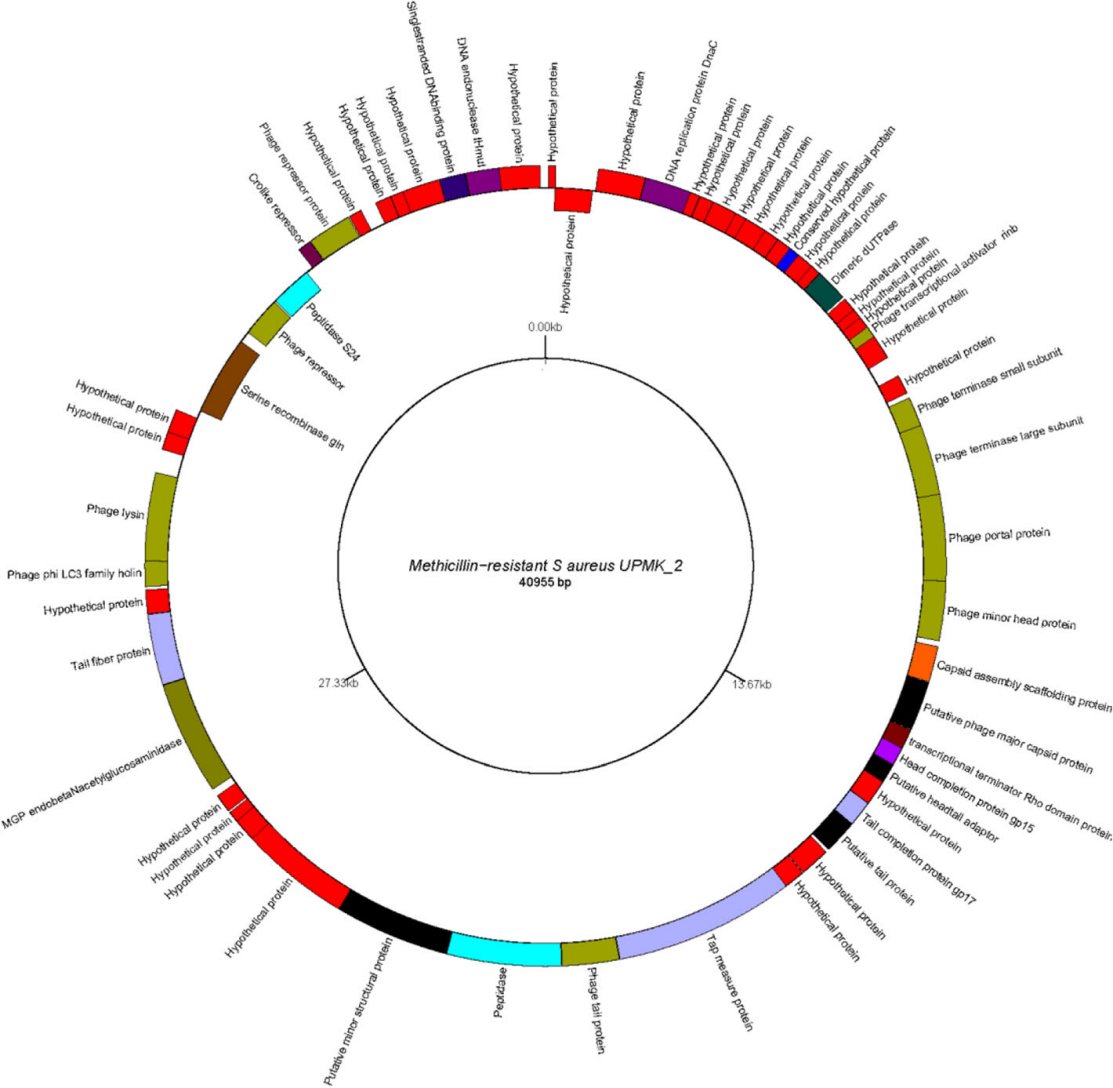
Genome map of UPMK_2 generated using GenomeVx available at http://wolfe.gen.tcd.ie/GenomeVx. Genes are shown as colored arcs and are labeled with the annotation proteins. Genes on outside are forward strand genes and genes inside are reverse strand genes

The next generation sequencing of UPMK_1 and UPMK_2 (dsDNA) genome was performed on the Illumina HiSeq 4000 platform. The UPMK_1 genome comprised 152,788 bp coding for 155 putative ORFs (Fig. 6; also refer to Additional file 3). The UPMK_1 has a low G + C content percentage of 31.9%, while UPMK_2 has a genome size of about 40,955 bp with 62 identified putative ORFs (Fig. 7; see also Additional file 4). UPMK_2 has a higher G + C content of 35.39% compared to UPMK_1. Most of the genes numbering 104 (67.1%) and 58 (93.5%) were found in the positive strand, and 51 (32.9%) and 4 (6.5%) were found in the opposite strand UPMK_1 and UPMK_2 respectively, while tRNAs genes were absent in both phages. In silico*,* the dot plot of the assembled contigs for phage UPMK_2 showed 140 bp overlapping features with a 0.0 mismatch (see the Additional file 5), these are characteristic features of a circular genome, whereas *Podoviridae* are traditionally known to have a linear genome but with few exceptions as reported for other *Podoviridae* phages with circular genome [37].

**Fig. 8:**
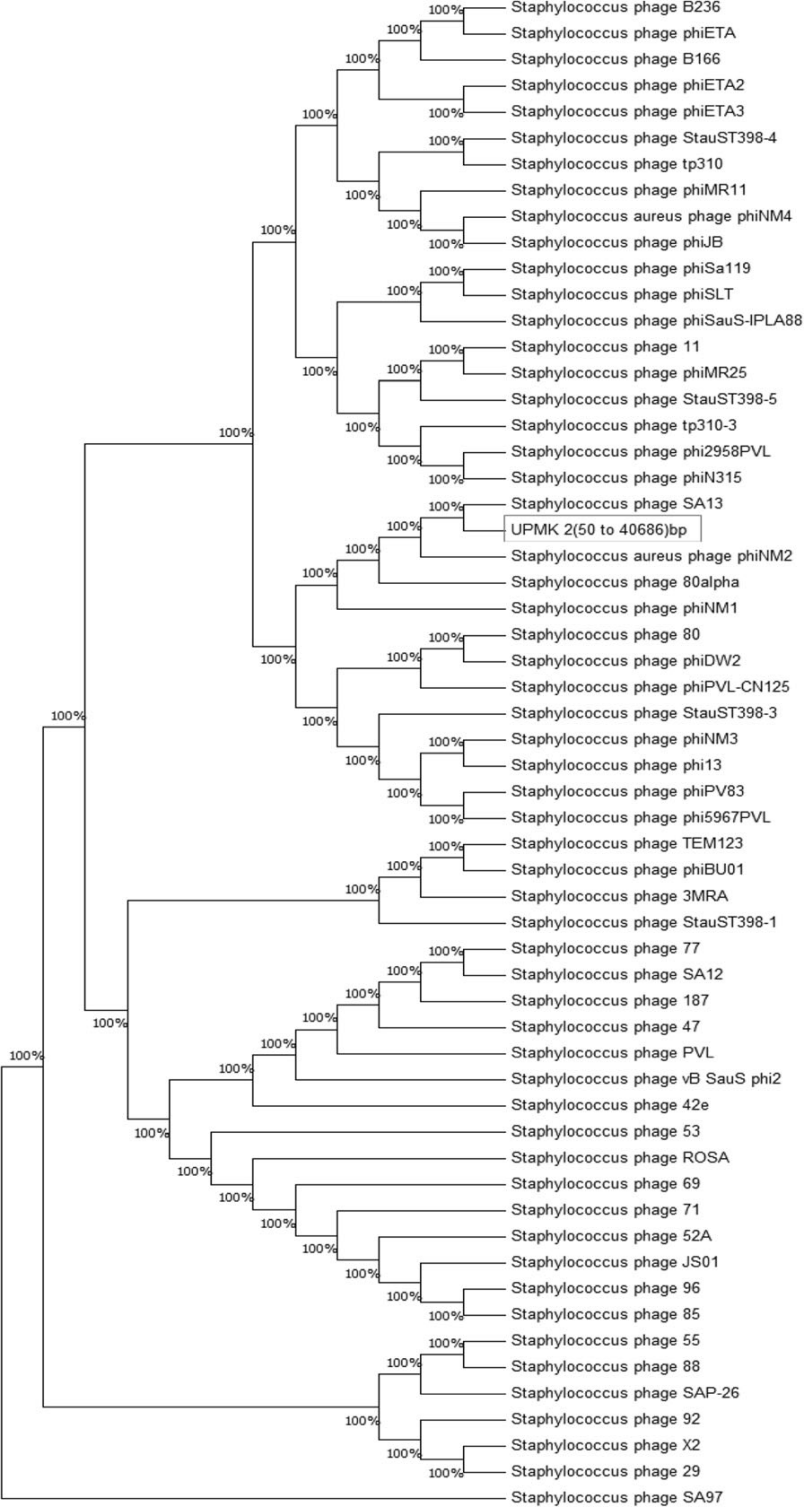
Phylogenic analysis showing the evolutionary history of the intact prophage that detected in the UPMK_2 genome by PHASTER analysis. The phylogenetic analyses were performed by using the maximum likelihood (ML) method based on the Tamura-Nei model. The tree with the highest log likelihood (− 3,039,991.6946) is shown. Initial tree(s) for the heuristic search were obtained automatically by applying Neighbor-Join and BioNJ algorithms to a matrix of pairwise distances estimated using the Maximum Composite Likelihood (MCL) approach, and then selecting the topology with superior log likelihood value. All positions containing gaps and missing data were eliminated. The analyses were conducted in MEGA7

**Fig. 9:**
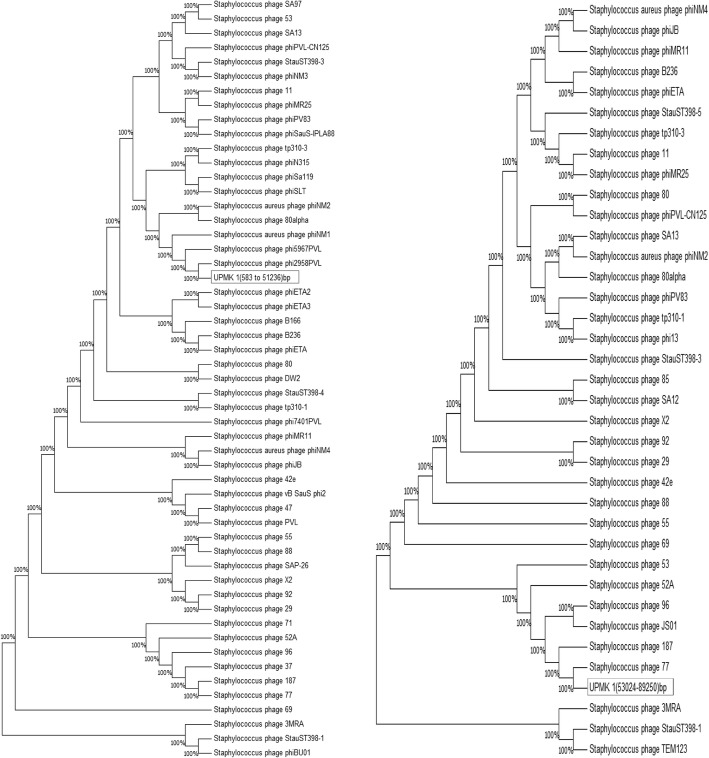
Phylogenic analysis showing the evolutionary history of the intact prophage (on the left) and incomplete prophage (on the right) that detected in the UPMK_1 genome by PHASTER analysis. The phylogenetic analyses were performed by using the maximum likelihood (ML) method based on the Tamura-Nei model. The tree with the highest log likelihood (− 2,768,365.1605) for intact prophage and (− 1,752,398.3348) for the incomplete prophage are shown. Initial tree(s) for the heuristic search were obtained automatically by applying Neighbor-Join and BioNJ algorithms to a matrix of pairwise distances estimated using the Maximum Composite Likelihood (MCL) approach, and then selecting the topology with superior log likelihood value. All positions containing gaps and missing data were eliminated. The analyses were conducted in MEGA7

The similarities of the nucleotide sequence of the isolated phage genome in this study with other bacteriophages were computed by sequence alignment (BLASTN) using default settings. It was observed from the obtained results that UPMK_1 showed a low similarity with other *Staphylococcus* phages of the *Myoviridae* family, as shown in Table 3. Furthermore, a low similarity was also observed between UPMK_2 and the *S. aureus* phages in the *Podoviriade* family such as phages S13’ (GenBank accession no. AB626963) and phage S24–1 (GenBank accession no. AB626962) with a query coverage of 2% and identity of 76%. While the similarity with phage SAP-2 (GenBank accession no. EU136189) gave a query coverage of 1% and an identity of 88%, phage 66 (GenBank accession no. AY954949) gave a query coverage of 1% and an identity of 76%. At the same time, PHASTER tools that were used to explore UPMK_1 and UPMK_2 genome showed that UPMK_1 contained two prophages; intact prophage region position (583–51,236) bp and incomplete prophage region position (53024–89,250) bp. The intact prophage gave the higher query coverage and similarity with 44 and 96%, respectively, with both of the StauST398_1 and StauST398_5 as shown in Table 4. While the incomplete prophage gave the higher query coverage of 27% and an identity of 99% with phage 92 as shown in Table 5. Whilst the PHASTER tools analysis uncovered one prophage region position (50–40,686) bp in the UPMK_2 genome, and BLASTN search (Table 6) showed it shared with phage SA13 the higher query coverage and similarity with 80 and 95%,respectively. Furthermore, maximum likelihood phylogenetic tree confirms the result of BLASTN in regards to UPMK_2 as shown in Fig. 8. In contrast, the maximum likelihood phylogenetic trees (Fig. 9) for intact and incomplete prophage in UPMK_1 fail to confirm the result of BLASTN. Inaccurate phylogenetic tree for intact and incomplete prophage in UPMK_1 may be due to a high frequency of gaps in its genome.

**Table 3:** Comparative nucleotide analysis between UPMK_1 and other members of *Staphylococcal Myoviridae* family using BLASTN

Phage	UPMK_1		Phage	UPMK_1	
	Query coverage	Similarity		Query coverage	Similarity
Twort	1%	67%	A5W	1%	67%
K	2%	67%	SA5	1%	67%
G1	1%	67%	S25–3	1%	67%
GH15	1%	67%	S25–4	1%	67%
SA11	2%	72%	P108	1%	67%
ISP	1%	67%	P68	1%	96%
676Z	1%	67%	Staphy1N	1%	67%
A3R	1%	67%	PSco-10	1%	100%
JD007	1%	68%	K/420	1%	81%
Romulus	2%	72%	Phi812	1%	67%
SEP1	2%	68%	Stau2	2%	77%
CIC	2%	68%	PhiSA039	1%	67%
RODI	2%	72%	pSa-3	1%	67%
SA012	1%	67%	MSA6	1%	67%
Sb −1	1%	67%	P4W	1%	67%
MCE-2014	1%	67%	SEP9	2%	70%
vB_SauM-fRuSau02	1%	67%	Team1	1%	67%

GenBank accession no.

Twort(AY954970),K(KF766114),G1(NC_007066),GH15(JQ686190),SA11(NC_019511),ISP(FR852584),676Z(JX080302),A3R(JX080301),JD007(JX878671),Romulus(JX846613),SEP1(KF021268),CIC(KP027447),RODI(KP027446),SA012(AB903967),Sb-1 (HQ163896),M CE-2014(KJ888149), vB_SauM-fRuSau02(MF398190),A5W(EU418428.1),SA5(JX875065),S25-3(AB853330) ,S25-4(AB853331),P108( KM216423.1),P68(NC_004679),PSco-10 (KX 01 10 28. 1 ), K/420 (KJ206563 ),Phi812(KJ206559), Stau2(KP8813321.1),PhiSA039 (AP0183 75.1),pSa-3(KY581279.1),Staphy1N (JX080300),MSA6(JX080304),P4W( JX080305), Team1(KC012913)

**Table 4:** Comparative nucleotide analysis between UPMK_1 region position (583–51,236) bp detected by PHASTER analysis as intact prophage (score > 90) and other members of *Staphylococcal siphoviridae* family using BLASTN

Phage	UPMK_1 (583–51,236)bp	
	Query coverage	Similarity
B236	39%	98%
Sap26	18%	98%
52A	41%	97%
phiETA	41%	96%
phiMR11	39%	96%
SA13	43%	96%
88	36%	96%
StauST398_1	44%	96%
StauST398_5	44%	96%
96	29%	89%
71	32%	97%
29	38%	96%
X2	35%	95%
phiPVL_CN125	11%	97%
80	42%	97%
55	41%	96%
PVL	7%	97%
phiPV83	11%	88%
phi2958PVL	6%	78%
phinm2	14%	94%
phiETA2	14%	94%
3MRA	27%	87%
92	36%	96%
phiJB	31%	88%
47	1%	95%
phi5967PVL	13%	92%
phiMR25	15%	94%
37	1%	71%
phiNM3	2%	97%
vB_SauS_phi2	2%	96%
phiNM	14%	94%
StauST398_3	23%	86%
tp310_1	6%	78%
Staphy_11	12%	96%
phiSa119	11%	90%
Lactob_Lj965	No significant similarity found	
Strept_phiNJ2	No significant similarity found	
42E	4%	96%
B166	26%	86%
tp310_3	6%	78%
phiN315	3%	96%
187	5%	93%
53	14%	94%
Slt	10%	93%
StauST398_4	2%	97%
phiSauS-IPLA88	12%	91%
DW2	25%	84%
Temper_phiNIH1.1	No significant similarity found	
77	6%	87%
phinm4	29%	88%
80alpha	13%	97%
phi7401PVL	5%	78%
Strept_315.4	No significant similarity found	
phiBU01	3%	93%
phiETA3	30%	88%
TEM123	10%	97%
Lactoc_TP901_1	No significant similarity found	
69	14%	96%
SA97	10%	96%

GenBank accession no.

52A(NC_007062),B236(NC_028915),phiMR11(NC_010147),Sap26(NC_014460),StauST398_1(NC_021326),phiETA(NC_003288),SA13(NC_021863),X2(NC_007065),88(NC_007063),StauST398_5(NC_023500),80(NC_030652),29(NC_007061),55(NC_007060),96(NC_007057),phiPVL_CN125(NC_012784),PVL(NC_002321),phiPV83(NC_002486),71(NC_007059),phiJB(NC_028669),phi2958PVL(NC_011344),phinm2(NC_028913),phiETA2(NC_008798),92(NC_007064),StauST398_4(NC_023499),47(NC_007054),phi5967PVL(NC_019921),phiMR25(NC_010808),37(NC_007055),phiNM3(NC_008617),3MRA(NC_028917),phiNM(NC_008583(2),StauST398_3(NC_021332),11(NC_004615),phiSa119(NC_025460),Lactob_Lj965(NC_005355),42E(NC_007052),B166(NC_028859),tp310_3(NC_009763),187(NC_007047),JS01(NC_021773),53(NC_007049),Slt(NC_002661),DW2(NC_024391),Temper_phiNIH1.1(NC_003157),77(NC_005356),phiNJ2(NC_019418),phiN315(NC_004740),phinm4(NC_028864),80alpha(NC_009526),phi7401PVL(NC_020199),Strept_315.4(NC_004587),vB_SauS_phi2(NC_028862),phiBU01(NC_026016),phiETA3(NC_008799),tp310_1(NC_009761),Lactoc_TP901_1(NC_002747),69(NC_007048),Ipla88(NC_011614),SA97(NC_029010)

**Table 5:** Comparative nucleotide analysis between UPMK_1 region position (53024–89,250) bp detected by PHASTER analysis as incomplete prophage (score < 70) and other members of *Staphylococcal siphoviridae* family using BLASTN

Phage	UPMK_1 (53024–89,250) bp	
	Query coverage	Similarity
92	27%	99%
88	18%	99%
53	18%	99%
85	25%	99%
Strept_9871	No significant similarity found	
phinm2	19%	96%
96	8%	93%
phiMR25	18%	97%
B236	16%	99%
11	14%	99%
69	16%	97%
42E	11%	98%
SA13	20%	99%
phiETA	6%	97%
phiJB	6%	98%
29	12%	98%
80alpha	17%	99%
55	13%	94%
phiMR11	13%	96%
StauST398_3	12%	88%
phiPV83	6%	95%
tp310_3	3%	97%
187	2%	91%
JS01	3%	92%
SA12	20%	99%
Staphy_StB20	No significant similarity found	
X2	17%	94%
phiPVL_CN125	2%	99%
77	5%	98%
phinm4	6%	98%
52A	13%	95%
13	4%	97%
3MRA	11%	95%
TEM123	10%	99%
Strept_9871	No significant similarity found	
Strept_9872	No significant similarity found	
StauST398_1	7%	90%
tp310_1	2%	97%
Staphy_80	13%	95%
Staphy_StauST398_5	7%	90%

GenBank accession no.

92(NC_007064),88(NC_007063),53(NC_007049),85(NC_007050),phinm2(NC_028913),phiMR25(NC_010808),69(NC_007048),96(NC_007057(3),80alpha(NC_009526),55(NC_007060),phiMR11(NC_010147),42E(NC_007052),80(NC_030652),SA13(NC_021863),phiJB(NC_028669),29(NC_007061),B236(NC_028915),11(NC_004615),StauST398_3(NC_021332),phiPV83(NC_002486),tp310_3(NC_009763),187(NC_007047),phiETA(NC_003288),JS01_NC_021773),SA12(NC_021801),StB20(NC_019915),X2(NC_007065),phiPVL_CN125(NC_012784),77(NC_005356(1),phinm4(NC_028864),52A(NC_007062),13(NC_004617),3MRA(NC_028917),Strept_9871(NC_031069),Strept_9872(NC_031094),TEM123(NC_017968),StauST398_1(NC_021326),tp310_1(NC_009761),StauST398_5(NC_023500)

**Table 6:** Comparative nucleotide analysis using BLASTN between UPMK_2 as intact prophage (score < 90) and most phages common hit (*Staphylococcal siphoviridae* members) detected by PHASTER analysis

Phage	UPMK_2	
	Query coverage	Similarity
88	69%	97%
92	78%	98%
B236	71%	97%
SA13	80%	95%
55	72%	95%
Sap26	35%	98%
phiMR11	68%	96%
96	52%	93%
StauST398_1	71%	94%
52A	71%	96%
29	67%	96%
X2	67%	93%
11	33%	98%
StauST398_5	71%	94%
80	73%	96%
53	39%	94%
phiETA	66%	95%
phiJB	53%	93%
phiMR25	40%	96%
phinm2	41%	96%
69	38%	96%
85	43%	99%
71	54%	97%
SA97	23%	98%
phiPVL_CN125	10%	97%
phinm4	48%	92%
phiETA2	29%	93%
StauST398_3	48%	88%
PVL	6%	97%
phiNM	29%	95%
42E	16%	98%
80alpha	37%	93%
tp310_3	10%	89%
phiPV83	14%	88%
77	13%	87%
phiNM3	4%	97%
phiSa119	12%	90%
187	8%	93%
JS01	5%	95%
StauST398_4	4%	97%
DW2	52%	85%
47	4%	95%
3MRA	53%	90%
phiETA3	51%	93%
B166	47%	89%
Strept_SM1	No significant similarity found	
SA12	37%	94%
Slt	11%	93%
phi2958PVL	5%	95%
phi5967PVL	14%	92%
Strept_315.2	No significant similarity found	
Strept_phiNJ2	No significant similarity found	
vB_SauS_phi2	10%	91%
Strept_9871	No significant similarity found	
Lactoc_TP901_1	No significant similarity found	
Temper_phiNIH1.1	No significant similarity found	
StB20	No significant similarity found	
phiN315	6%	96%
13	12	92
Strept_phiARI0923	No significant similarity found	
Strept_315.4	No significant similarity found	
ROSA	48%	93%
Strept_9872	No significant similarity found	
Staphy_TEM123	28%	92%
tp310_1	5%	97%
Ipla88	31%	88%

Interestingly, phage UPMK_1 contained type I restriction-modification system subunit M (*orf*75) and specificity determinant *hsdS* (*orf*76) which will have a marked effect on the EOP of the bacteriophage [38, 39]. This observation was based on the phage growth cycle in certain hosts which exerts a certain effect on the ability of the phage progeny to grow by restricting their bacterial host range to only the bacteria that have same R-M system in the UPMK_1. The poor EOP of UPMK_1 when cultured with different isolates of MRSA was observed to have a high ability to propagate only on the MRSAt127/4 with a low observable incidence of propagation on other MRSA isolates.

**Fig. 10:**
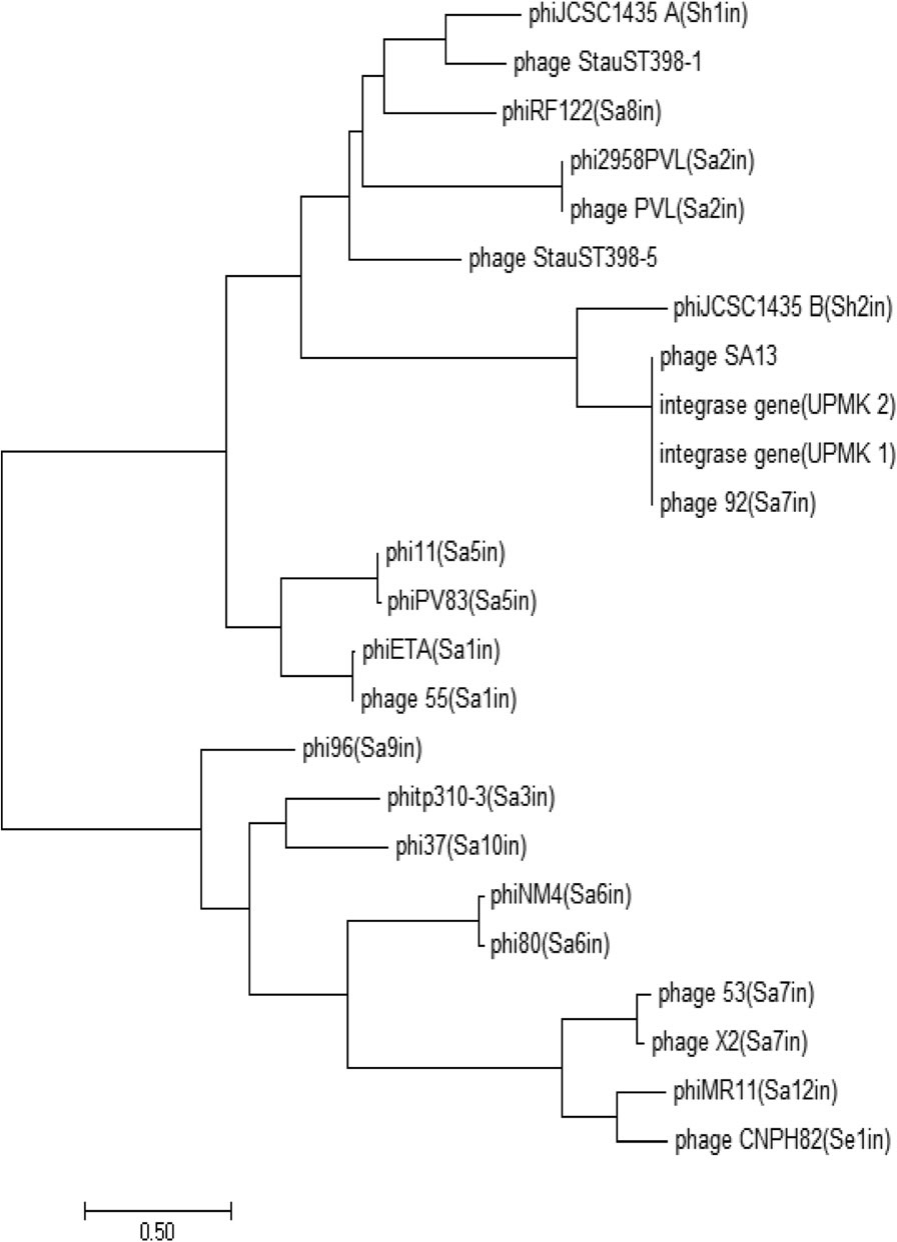
Molecular phylogenetic analysis of phage integrase gene sequence by Maximum Likelihood method based on the Tamura-Nei model. The tree with the highest log likelihood (− 15,349.6750) is shown. Initial tree(s) for the heuristic search were obtained automatically by applying Neighbor-Join and BioNJ algorithms to a matrix of pairwise distances estimated using the Maximum Composite Likelihood (MCL) approach, and then selecting the topology with superior log likelihood value. The tree is drawn to scale, with branch lengths measured in the number of substitutions per site. The analysis involved 24 nucleotide sequences. Codon positions included were 1st + 2nd + 3rd + Noncoding. All positions containing gaps and missing data were eliminated. Evolutionary analyses were conducted in MEGA7

Further analysis of the genome annotation revealed that UPMK_1 and UPMK_2 had a lysogeny module. The classification depended on integrase gene variety (Fig. 10) within the integrase cluster of UPMK_1 and UPMK_2 with integrase gene of phage 92 and phage A13 sharing the same node with phi JCSC1435 B integrase group Sh2 . This integrase is related to serine recombinase-type family. Therefore, both phages may also have a gene that could be responsible for the inactivation of the transcription repressor, as represented by anti-repressor gene orf138 in UPMK_1 and Cro-like repressor (orf 54) in UPMK_2.

A conserved domain search using the NCBI’s BLASTN/BLASTP (default search parameters) for individual proteins showed that UPMK_1 contained two endolysin-like proteins, with their holin compared to UPMK_2. The first endolysin in UPMK_1 (represented by *orf* 81) has the same structure as the endolysin in UPMK_2 (*orf*48) by having three domains (a CHAP domain, a peptidoglycan recognition proteins PGRP (amidase-2 domain), and a SH3–5 domain). The second endolysin (represented by *orf*114 in UPMK_1) was found to contain the CHAP domain and amidase-3. Furthermore, UPMK_2 contained *orf*44 which has two catalytic domains (CHAP domain and glucosaminidase domain).

Apart from the endolysin-like proteins, UPMK_1 also contained dipeptidyl aminopeptidase which represents *orf*57 with peptidase_S9 (Pfam00326) active domain. This active domain belongs to the Abhydrolase superfamily, and could be considered as a part of the xylanolytic system that hydrolyzes xylan, a type of hemicelluloses [40]. Interestingly, the *orf*53 with 418–621 aa in UPMK_1 seemed to be similar to the *orf*38 with 418–623 aa in UPMK_2. Both sequences were considered as conserved domains of a protein belonging to the SGNH_hydrolase. This enzyme contains lipase_GDSL-2 and esterase domains. These conserved domains had a 99% identity with a query coverage of 100% to the tail endopeptidase (*Staphylococcus* phage B236, accession number: YP009209179.1), and phage minor structure protein (accession number: WP016187667.1). Previous reports have demonstrated that the *Phietalike* viruses within the *Siphoviridae* family contain SGNH proteins that are involved in the hydrolysis of fatty acids, aromatic esters, and amino acid derivatives [41].

### Phage lytic assessment on dispersal and degradation the biofilm

**Fig. 11:**
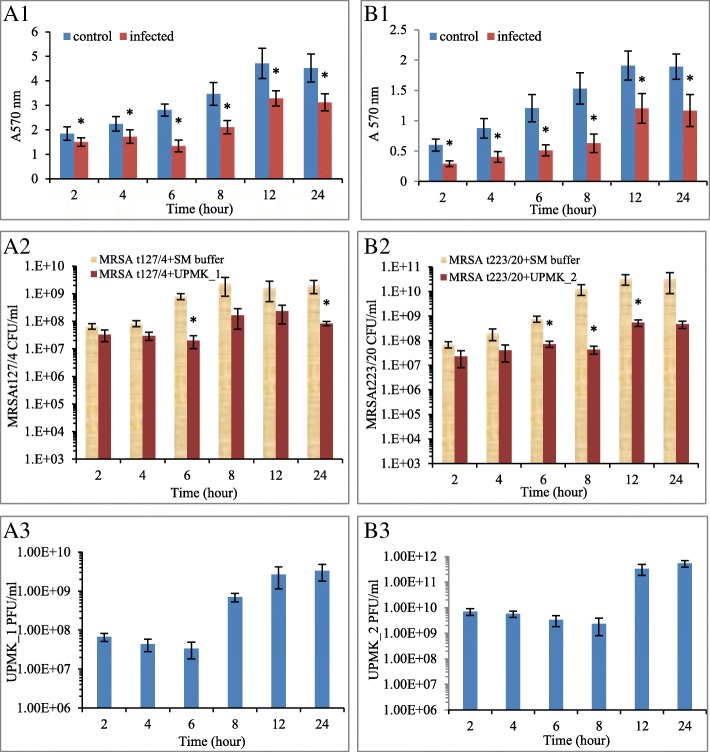
MRSA t127/4 and MRSA t223/20 biofilms treated with UPMK_1 and UPMK_2 for 2, 4, 6,8,12 and 24 h. A1 and B1 are biofilm biomass treated with the phages at MOI ~ 1 (OD570 reading after CV staining). The estimated number of viable bacterial cells is shown in A2 and B2, while A3 and B3 represent phage particles in the biofilms treated with UPMK_1 for 6 h and with UPMK_2 for 8 h, respectively. The assays were performed severally to assess the ability of the phages to degrade the biofilm, and thrice to estimate the other values. The values are shown as the mean ± standard deviation of the values; statistical significance of biofilm reduction was reported at *p* < 0.05

**Fig. 12:**
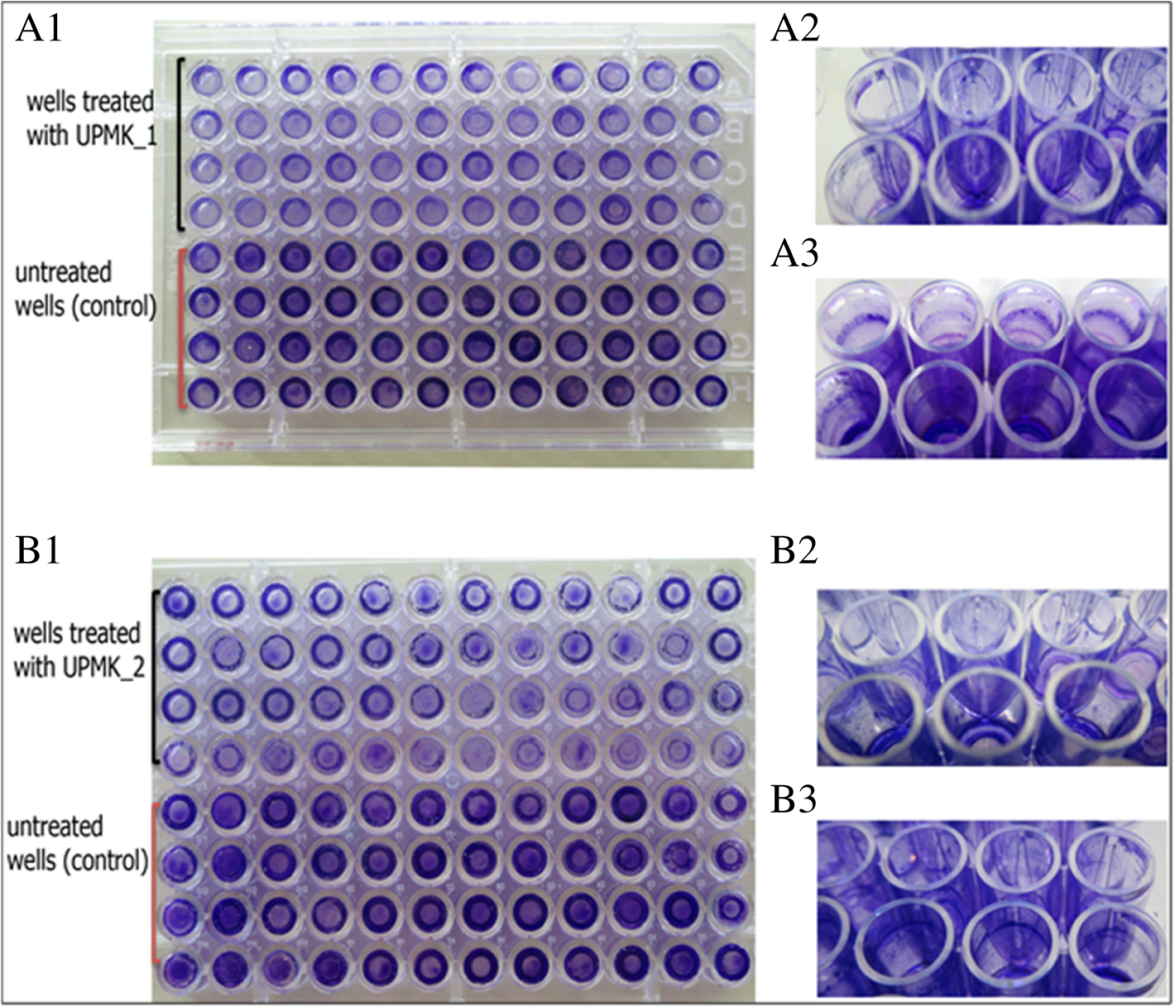
Tissue culture plates (TCP) of of MRS t127/4 and MRSA t223/20 biofilms which achieved a maximum biofilm removel after treatment with UPMK_1 and UPMK_2 FOR 6 h (A1) and 8 h (B1), respectively. (A1) and (B1) represent the bottom of the TCP, (A2) and (B2) represent the side view of the wells after treatment with the phages, and (A3) and (B3) represent the side view of the wells before treatment with the phages (control)

In order to assess the ability of the phages to disperse and degrade biofilms, 48 h-old biofilms which were produced by MRSA t127/4 and MRSAt223/20 isolates were seeded in 96-well microtiter plates. Although MRSA t127/4 was considered as a moderate biofilm producer while MRSA t223/20 was considered as a weak biofilm producer [42], both biofilms were structurally rigid. They were more stable at the bottom and sides of the microtiter plate wells when performing the washing steps. The established biofilms were treated with phage suspensions at an MOI of 1 and assessed after 2, 4, 6, 8, 12, and 24 h by crystal violet staining (Figs. 11 A1 and B1). The results showed that there was a clear reduction following phage inoculation compared to the non-phage inoculated wells. A significant decrease in the biofilms was physically observed along the CV stained wells (Fig. 12). UPMK_1 showed a gradual reduction of the biofilm biomass after 2 h (18.6%), followed by 23% reduction after 4 h, and reaching the maximum of 52% reduction after 6 h. Although the biofilm biomass remained lower at 12 and 24 h when compared to the untreated biofilms, the process of biofilm regrowth was continual after 8 h and 12 h. UPMK_2 showed a 51% biofilm biomass reduction within 2 h after phage inoculation which was, compared to UPMK_1. After 8 h of treatment, more than 58% of the biofilm biomass was eliminated by UPMK_2; thereafter, the observed biofilm regrowth was constant after 12 and 24 h compared to the regrowth after 8 h. The measured biofilm biomass after 24 h was lower than the control biofilm biomass. A similar behavior was observed following the disruption of the biofilms after phage treatment as both bacterial and phage numbers were estimated. For MRSA t127/4, after 6 h of treatment, the number of cells was observed to reduce 2 logs with a *p* value of 0.003 (Fig. 11 A2). This was followed by a regrowth after 8 and 12 h. By the end of 24 h, a reduction in the cell number was observed at the rate of 1 log with a *p* value of 0.02. However, the same trend was observed for the disrupted biofilm (Fig. 11 A1), which could be related to the activity of the phage within the bacteria since the bacteria within the biofilms were found to be in a different metabolic state. The number of phages in the biofilm was observed to decrease in concentration after 6 h most likely due to the absorption of most of the phages into the bacteria. Subsequent observations showed an increase in the phage number with time (Fig. 11 A3). Similarly, there was a 3 log reduction in the number of viable cells for MRSA t223/20 after 8 h of treatment (*p* = 0.02; Fig. 12 B2), followed by a regrowth after 12 and 24 h. Regarding the phage numbers in the biofilm, it was observed that after 8 h, there was a decrease in the phage concentration, followed by an increase after 12 and 24 h (Fig. 11 B2). Such an observation supports the fact that the phages were actively replicating and that biofilm reduction and dispersion were a consequence of the phage addition and infection.

**Fig. 13:**
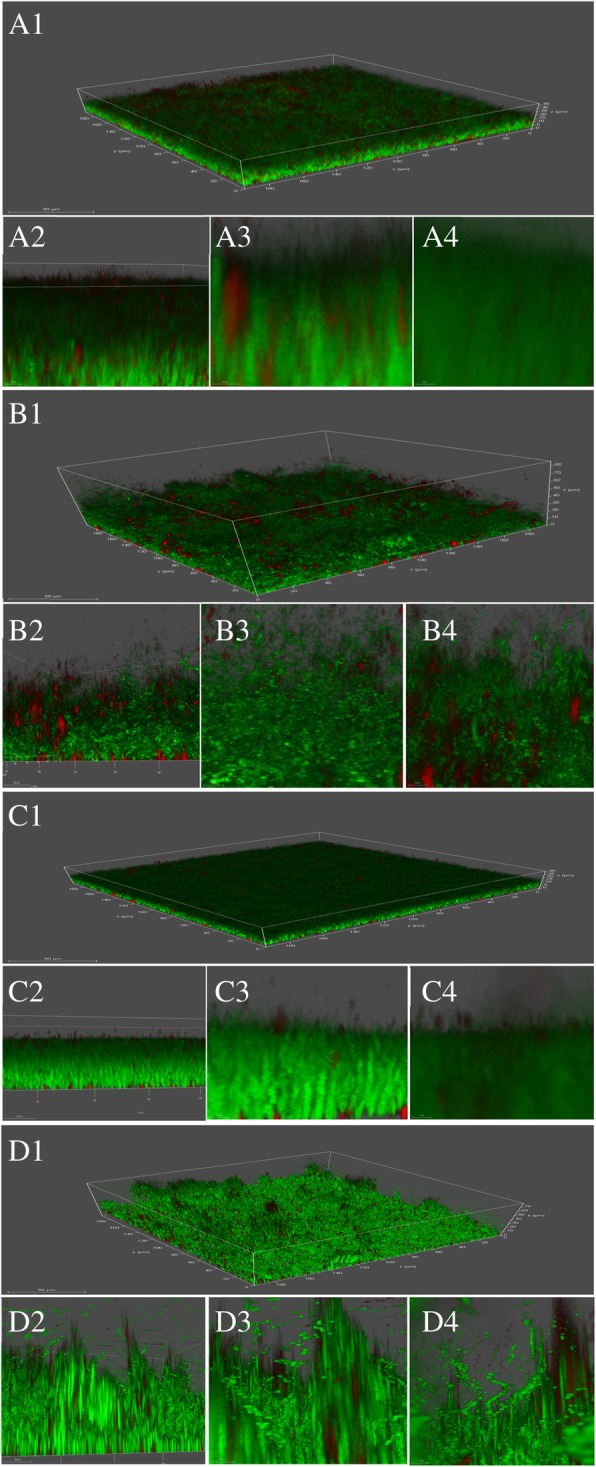
CLSM micrographs of the biofilms formed by MRSA before and after treated with bacteriophage. A1 (scale bar; 50 μm), A2 (scale bar; 20 μm), A3 (scale bar; 10 μm) and A4 (scale bar; 5 μm) were represented the biofilm of MRSAt127/4 that treated with SM buffer as control. B1 (scale bar; 50 μm), B2 (scale bar; 20 μm), B3 (scale bar; 10 μm) and B4 (scale bar; 5 μm) were represented the biofilm of MRSAt127/4 that treated with phage UPMK_1 for 6 h. C1 (scale bar; 50 μm), C2 (scale bar; 20 μm), C3 (scale bar 10 μm) and C4 (scale bar; 5 μm) were represented the biofilm of MRSAt223/20 that treated with SM buffer as control. D1 (scale bar; 50 μm), D2 (scale bar 20 μm), D3 (scale bar 10 μm) and D4 (scale bar; 5 μm) represents the biofilm of MRSAt223/20 that treated with phage UPMK_2 for 8 h. Three dimensional projections of biofilm structure were reconstructed using Lecia LAX software

For the in situ visualization of the biofilms in their natural hydrated states and to quantitatively assess the structural changes induced by the phages, CLSM, coupled with Filmtracer LIVE/DEAD biofilm staining were performed [43–45]. The times needed to achieve the maximum biomass reduction using UPMK_1 and UPMK_2 were evaluated within 6 h and 8 h, respectively. A 48 h-old biofilm of MRSA t127/4 and MRSA t223/20 was grown on modified cell culture slides before treatment for 6 and 8 h with UPMK_1 and UPMK_2 at an MOI ~ 1, respectively. The eradication and dispersion of the biofilms were then assessed via the visualization of a stained specimen under confocal microscopy. A clear reduction of the biofilms was observed. The dispersed bacterial aggregates which attached to the glass coverslip were compared to the ones treated with SM buffer (control), and disruption was confirmed by Lecia software parameters. The relationship between the volume percentage and mean intensity for control and treated biofilms was low, and the low volume percentage of the biofilm which is linked to the high mean of intensity was a good pointer to the disruption of the biofilm by the phages compared to that of the control (as reflected by the compact structure of the biofilm which had a high-volume percentage with low intensity) (Fig. 13, Table 7).

**Table 7:** Distribution of values for both green and red channels obtained from CLSM and Leica LASX software analysis. These values confirmed biofilm disruption. The biofilm volume percentage for the control was higher than that of the treated biofilms, indicting biofilm disruption by the phages

Statistics	Volume Percent [%]	Volume [μm^3^]	Intensity Sum	Intensity Mean	Intensity Standard Deviation
MRSAt127/4 (control)					
Trial (1)	40.67	629,162.26	6,964,491,492	116.63	52.39
Trial (2)	92.18	1,303,588.41	10,599,441,732	85.66	61.65
Trial (3)	98.94	1,300,775.17	5,824,474,595	47.17	48.10
MRSA t127/4 treated with UPMK_1 for 6 h					
Trial (1)	4.72	152,555.31	1,910,159,851	131.92	53.22
Trial (2)	7.18	148,423.93	2,142,157,850	152.06	43.82
Trial (3)	11.18	132,164.16	1,793,990,059	143.01	46.24
MRSAt223/20 (control)					
Trial (1)	29.96	629,075.11	5,126,536,159	85.86	32.23
Trial (2)	21.85	333,184.01	4,568,795,881	144.47	60.08
Trial (3)	23.70	222,606.61	2,746,429,683	129.99	44.63
MRSA t223/20 treated with UPMK_2 for 8 h					
Trial (1)	9.13	277,600.54	3,511,210,967	133.26	45.45
Trial (2)	8.26	429,907.84	6,218,234,982	152.39	61.49
Trial (3)	9.13	115,041.66	1,442,935,232	132.15	46.90

## Discussion

Failure in antibiotic therapy especially related to bacterial biofilm formation and the emergence of MRSA are continuing major challenges in the treatment of chronic infections [46]. The use of phages and their enzymes can serve as alternative therapeutic strategies in treating biofilm-associated infections. Although bacterial biofilms are ubiquitous in nature [47], it is still unclear whether phages with bactericidal activity occur widely in nature. Therefore, this study focused on the isolation and characterization of bacteriophages against clinically isolated MRSA biofilm producers.

In this study, two phages (UPMK_1 and UPMK_2) were isolated. While phages have been suggested to be the predominant lifeforms in the biosphere [48, 49], MRSA-specific phages are rare. In the case of the biofilm-producing MRSA, it is more difficult to obtain their specific phages as they will be adsorbed into the dead cells and cell debris within the biofilm. These barriers hinder phage diffusion, especially phages lacking in peptidoglycan hydrolase activity which degrades extracellular substrate materials and facilitates phage diffusion and infection. Some studies reported that phage T7 and PRD1 mutant phages without virion-associated peptidoglycan hydrolase activity can infect host cells but the process is significantly delayed [50, 51].

The plaques produced by UPMK_1 and UPMK_2 were generally small and surrounded by turbid halos. The presence of halos around the clear plaque zone is a good indication that the diffusion of the phage enzymatic molecules (such as EPS depolymerases) can be efficient in dispersing biofilms. These halo zones increased with time as shown in Fig. 2 D, E and I. These proteins are much smaller than a whole phage; hence, they diffused at a higher rate into the bacterial lawn and caused the degradation of bacterial produced polymers. These phages have biotechnological applications in the treatment or control of infectious biofilms due to their ability to produce (or to be able to induce) enzymes that can degrade extracellular matrix [52].

Both UPMK_1 and UPMK_2 were shown to have a low adsorption rate, which in biofilm-like environment, is of great advantage compared to a high adsorption rate. Gallet et al. [47] reported the beneficial properties associated with phage settlement having a high adsorption rate, and with a consequent detrimental effect on phage production, particularly as it affects plaque size and productivity, as well as emigration efficiency. Similarly, a high adsorption rate during settlement can in most instances be compensated with the disadvantages associated with the production and emigration stages. It looks like that low adsorption rate for UPMK_1 and UPMK_2 could be as an adaptation for phage habitats, leading to an enhanced phage bacterial infection in either a planktonic or biofilm habitat.

At the genome level, UPMK_1 has a large double stranded DNA (dsDNA) like other members of the *Myoviridae* family with genome size > 125 kb [53] and low GC containing two endolysins. It also contains the gene of virility like other members of the *Siphoviridae* family [54]. UPMK_2 has a large dsDNA when compared to the genome size of the *S. aureus Podoviridae* phages [53, 55], but is comparable in genome size to the *Podoviridae* (40 to 42 kb). Important information on the functions and evolutionary relationships shared among several phage genomes could be deduced from the genome comparison of UPMK_1 and UPMK_2 with other *S. aureus* phages. Based on the functional conserved sequence, an annotation of the UPMK_1 and UPMK_2 structural genes were done and the comparison of the phage genes with other bacteriophages revealed shared identities in terms of function. However, based on the genome functional modules, both UPMK_1 and UPMK_2 showed minimal homology to other *S. aureus*-related bacteriophages. Furthermore, the PHASTER bioinformatics tool showed that both UPMK_1 and UPMK_2 have intact prophage showing high similarity with low query coverage. In addition, the UPMK_1 and UPMK_2 genomes are punctuated by a large number of inserted genes. This indicates sequence diversity in terms of rearrangements, insertions, or losses which could be a major mode of bacteriophage evolution. Such a mosaic genomic sequence variation may result from productive recombination events. The modular theory of phage evolution and diversity may also explain the potential interaction between the infecting phage and other prophages in the host that may lead to the development of hybrid phages.

It was not possible to assign UPMK_1 to a particular family based on the genomic sequencing. Its genomic characteristics and phage morphology were between those of *Siphoviridae* and *Myoviridae*. Based on the TEM morphological features, UPMK_2 was assigned to the *Podoviridae* family but the genomic sequence did not show any similarity with any of the *S. aureus* phages related to the *Podoviridae* family. The presence of lysogeny module in both phages was not expected as this makes them unsuitable as candidates for phage therapy; with that, both phages presented a high lytic activity against their hosts. Further, UPMK_2 showed a high lytic ability against other MRSA isolates. It is possible that these phages had undergone mutations to assume a lytic ability, but further investigations are required to confirm this. The development of phage or phage-based products can contribute immensely to genome sequencing in the genetic modification of phages.

Characterization and genomic sequencing can further elucidate the functional encoded genes and their roles in phage replication properties, as this can be clearly observed between the EOP, type I restriction-modification system subunit M, and specificity determinant *hsdS* genes in UPMK_1. This made UPMK_ 1 limited in its host range. The activity of UPMK_1 and UPMK_2 against the 25 MRSA isolates showed differences in phage productivity although a majority of the isolates were related to the same *spa* type and biofilm matrix, hence closely related. Bull et al. [56] suggested that partial rather than absolute resistance may be causing different productivity to the standard models (bacteria with high productivity and no productivity). This indicates that the bacteria were partially resistant to the bacteriophage due to phenotypic resistance rather than genetic.

‘Lysis from without’ is a complex process and UPMK_2 has exhibited this process towards all MRSA (ST239) isolates. This could be either due to the activity of the cell wall hydrolase (*orf*43) and peptidase (*orf*37) which can lyse the cells without propagating the phage in the bacteria or due to the secondary adsorption of the phage to an already phage infected bacterium. Therefore, this may not show up in a plaque count assay, indicating the loss of the virion. These phages will moreover, interfere with the phage growth in these bacteria and cases bacteria lysis without phage propagation [57]. This interference between mixed phages and bacteria which can lead to ‘lysis from within’ or ‘lysis from without’ is of great advantage as it will often result in the lysis of the bacteria.

In vitro two biofilm models were employed to study biofilm reduction and dispersion by phage attack. Both UPMK_1 and UPMK_2 decreased the biofilm biomass in the microtiter assay, though a complete elimination of biofilm was not observed even after 24-h of treatment. As the biofilm ages, there was a proportionate increase in the ratio of dead to live cells within the biomass. This makes the complete eradication of the cells in the biofilm more difficult because phages bind equally to receptor sites on both living and dead cells. This effectively reduces their viable cells infection rate [58]. In addition, biofilm contained several subpopulations with different phenotypic and metabolic properties. Consequently, these subpopulation of bacteria may appear to be resistant toward phages that are associated with lysogeny, restriction modification, mutations that affect phage adsorption, and various defense genes carried by plasmids or prophages or the mechanisms of abortive infection such as the presence of clustered regularly interspaced short palindromic repeats (CRISPRs) in the bacterial genome [59–61]. Furthermore, the bacterial metabolism in older biofilms continued to decrease at a lower rate, especially when the bacteria were present in the deeper layers of the biofilms where oxygen and nutrient availability are limited. As phage infection and phage life cycle are both dependent on the growth stage of the host cell [62], Azeredo and Sutherland [63] claimed that the more slowly-metabolizing cells present in the older biofilms would demonstrate an increased degree of phage resistance. Lenski [64] has claimed that in a closed system during phage-host interaction, the co-evolutionary potential of phage is less than their bacterial host strains, allowing bacterial mutants with no corresponding lytic phage strain to evolve. Since the reduction in the 24 h biofilm biomass in this study was comparable to that of a 12 h biofilm after phage treatment (Fig. 12 A1 and B1), the concentration of phages increased (Fig. 12 A3 and B3) without completely decreasing the bacterial number after 12 and 24 h (Fig. 12 A2 and B2). The increase in the phage titer reflected the replication and propagation of the phages within the biofilm. These results were compatible with previous phage therapy studies that examined the post-treatment phage concentrations [65]. The observed biofilm disruption in microtiter plates after 6 h and 8 h for both MRSAt127/4 and MRSA t223/20 (Fig. 12 A1 and B1), as confirmed with confocal laser scanning microscopy images showed that the phages completely disrupted the bacteria compared to the control; and that the dense structure of the biofilms were loose as the bacteria began to differentiate and emerge from the biofilms. The emergence of the bacteria from the biofilms related to the biofilm detachment process. With that, the bacteria can slow down the propagation of the phages and thus, delay the death of the biofilms [66]. Although a fraction of the bacteria can evade phage attack, this mechanism cannot completely prevent phage replication. The propagation of phages in the biofilm (Fig. 12 A3 and B3) illustrated their ability to infect cells, replicate within them, and lyse them, though the bacterial cells were not completely eliminated (Fig. 12 A2 and B2). The regrowth of the biofilm after 12 h (Fig. 12 A1 and B1) may be a consequence of the slow metabolic activity of the bacteria in the biofilm, followed by the slowed phage proliferation process which results in the emergence of resistant phage clones in the bacterial population [67, 68].

## Conclusion

Two phages (UPMK_1 and UPMK_2) with a high biological diversity were isolated and characterized based on biological (plaque size and morphology, one-step growth, and host range) and morphological (electron microscopic analyses) features. Genome sequence analysis of these isolated phages revealed the presence of virion-associated enzymes which are considered crucial in the degradation of the biofilms. Both microtiter plate and CLSM analyses confirmed the dispersion of MRSA biofilm biomass by these two isolated phages. Their virion enzymes are the key players in changing the adhesive phenotypic biofilm properties of bacterial cells by attaching to the biofilm matrix and facilitating both biofilm degradation and phage infection. The presence of these enzymes made UPMK_1 and UPMK_2 and their products investigable for biofilm dispersion.

## Methods

### Bacterial strains used for phage isolation

Twenty-five (25) isolates of biofilm-producing MRSA (t127/1, t127/4, t127/6, t127/7, t127/8, t2246/9, t127/14, t127/17, t127/18, t790/19, t223/20, t127/22, and t127/25 that formed PIA-dependent biofilms t127/2, t127/3, t127/5, t127/10, t127/11, t127/12, t127/13, t127/15, t127/16, t127/21, t127/23, and t127/24 that formed PIA-independent biofilms) were characterized [42] and used for the isolation and proliferation of MRSA lytic phages.

### Isolation of phages

Fifty samples were collected from different sources (sewage treatment plants from different colleges of Universiti Putra Malaysia (UPM), sea water (Port Dickson, Malaysia), UPM park lakes and swimming pool water, and poultry droppings around the hostel facilities of the Faculty of Veterinary Medicine, UPM) and soaked in salt of magnesium (SM) buffer (99 mM NaCl, 16 mM MgSo_4_, 50 mM Tris HCl pH 7.4 and 0.01% gelatin per 1 L of dH_2_O) overnight. The samples were centrifuged at 3000×g for 30 min and filtered through 0.45 μm syringe filter units (Millipore, USA). Then, 10 mL of the filtrates were mixed with 5 mL of 2x Tryptone Soy Broth (TSB), 5 mL of MRSA suspension in mid-exponential log phase, and CaCl_2_ to a final concentration of 7 mM. The reaction mixtures were incubated for 24 h at 37 °C in a 50-mL falcon tube in a rotary shaker at 80 rpm. The cultures were clarified via a three-step centrifugation process at 3000×g for 3 min, then, 5000×g for 3 min, and finally for 5 min at 8000×g. The supernatants were filtered with 0.45 μm filter to remove unwanted bacterial cells and used to check for the presence of specific MRSA phages using the overlay method [69]. To increase the possibility of isolating MRSA phages, both molten agar (0.6% agar per 100 mL TSB) and basic media (TSA) in plates were supplemented with 7 mM CaCl_2_ and 1 mM MgSO_4_, respectively. The isolated phages were further purified using a streak plating method described by phage hunting protocols [70].

### Concentration of phage stocks by polyethylene glycol (PEG) precipitation

The adopted protocol was modified from a standard protocol described by [71]. A single colony of the MRSA host strain in 10 mL of TSB broth was incubated overnight at 37 °C. The overnight growth culture (1 mL) was inoculated into 500 mL of TSB broth in 1 L screw cap flasks and incubated at 37 °C in a rotary shaker at 150 rpm. On reaching the mid-exponential phase, the growth culture was supplemented with 7 mM CaCl_2_ and inoculated with 1 mL of PFU/mL and incubated at 37 °C for 12 h. The cultures were brought to room temperature after 12 h incubation and treated with DNase and RNase (10 U/mL), mixed, and further incubated for 1 h at 37 °C. To each 500 mL of culture, NaCl was added (final concentration 1 M) and mixed gently until completely dissolved. The cultures were placed on ice for 1 h, followed by centrifugation, before filtering the phage lysates through a 0.45 μm nylon membrane filter by using bottle top filter Nalgene (Thermo scientific, UK).

The polyethylene glycol (PEG 8000) solution was added to the filtrates to a final concentration of 8.6% and gently mixed before incubating overnight at 4 °C. The phage particles were recovered by centrifuging the filtrates at 20,000×g for 6 h at 4 °C. The supernatant was discarded and 500 μL of SM buffer was added to the phage pellets and gently resuspended after overnight incubation at 4 °C. The phage suspensions were collected, and PEG was removed by adding 1 M KCl, and incubating on ice for 15 min before centrifuging at 8000×g for 10 min. The titer of the recovered phages in the supernatant was determined and used in performing the other assays in this study.

### Phage lytic assessment and sensitivity screening

Planktonic bacterial cultures were used for the lytic assessment. A single colony of host MRSA bacteria was inoculated into TSB and incubated overnight. The overnight culture (0.5 mL) was transferred into 50 mL of TSB and incubated to achieve an OD_600_ of 0.8 ~ (1 × 10^9^ CFU/mL). The culture was treated with 0.5 mL of the phage sample (10^11^ PFU/mL) before adding CaCl_2_ (7 mM) to achieve MOI ~ 1. All the culture mixtures were incubated in a shaker at 80 rpm at 37 °C for 12 h. The phage lytic assay was conducted in triplicates for each phage. The sensitivity screening was done based on the spot method using the previously characterized MRSA biofilm producers [42]. The host range was determined on MRSA strains related to ST239 which was obtained from the Medical Microbiology Laboratory of UPM [72]. About 300 μL of the log phase culture of the bacteria was added to 3 mL of soft-agar supplemented with 7 mM CaCl2 and allowed to solidify. Both phage samples were diluted to a titer of 1 × 10^7^ PFU/mL. Bacterial lawns were spotted with 10 μL of each phage lysate. The assay was done in triplicates and the plates were incubated overnight at 37 °C. Similarly, the efficiency of plating (EOP) was estimated by obtaining the ratio of the bacteriophage plaque titers from the different host (25 MRSA biofilm producers) to host used in the phage amplification.

### Adsorption and growth rate determination of phage

The adsorption efficiency of the phage in its host was measured by titrating free unabsorbed phages present in the supernatant after a defined period of phage/bacteria contact [73]. Overnight culture (0.1 mL) was used to prepare host MRSA cells in a 10 mL TSB medium. This was incubated at 37 °C with shaking at 180 rpm until OD_600_ = 0.8 ~ (10^9^ CFU/mL) was achieved. Then, the phage lysate was added into the bacterial culture to achieve an MOI = 0.01 with CaCl_2_ (final concentration 7 mM), and incubated with shaking at 80 rpm at 37 °C. One milliliter of the samples (in 1.5 microcentrifuge tube) was removed at 0, 3, 6, 9, 12, and 15 min intervals and immediately centrifuged for 3 min at 3000 x g at 4 °C to isolate and enumerate the unabsorbed phages in the supernatant. The samples were measured in triplicates. Furthermore, the one-step growth curve of the phages was done based on the protocol described by Carlson [74] with the MOI modified to 0.01 instead of 1.

### Purification of the phages

**Fig. 14:**
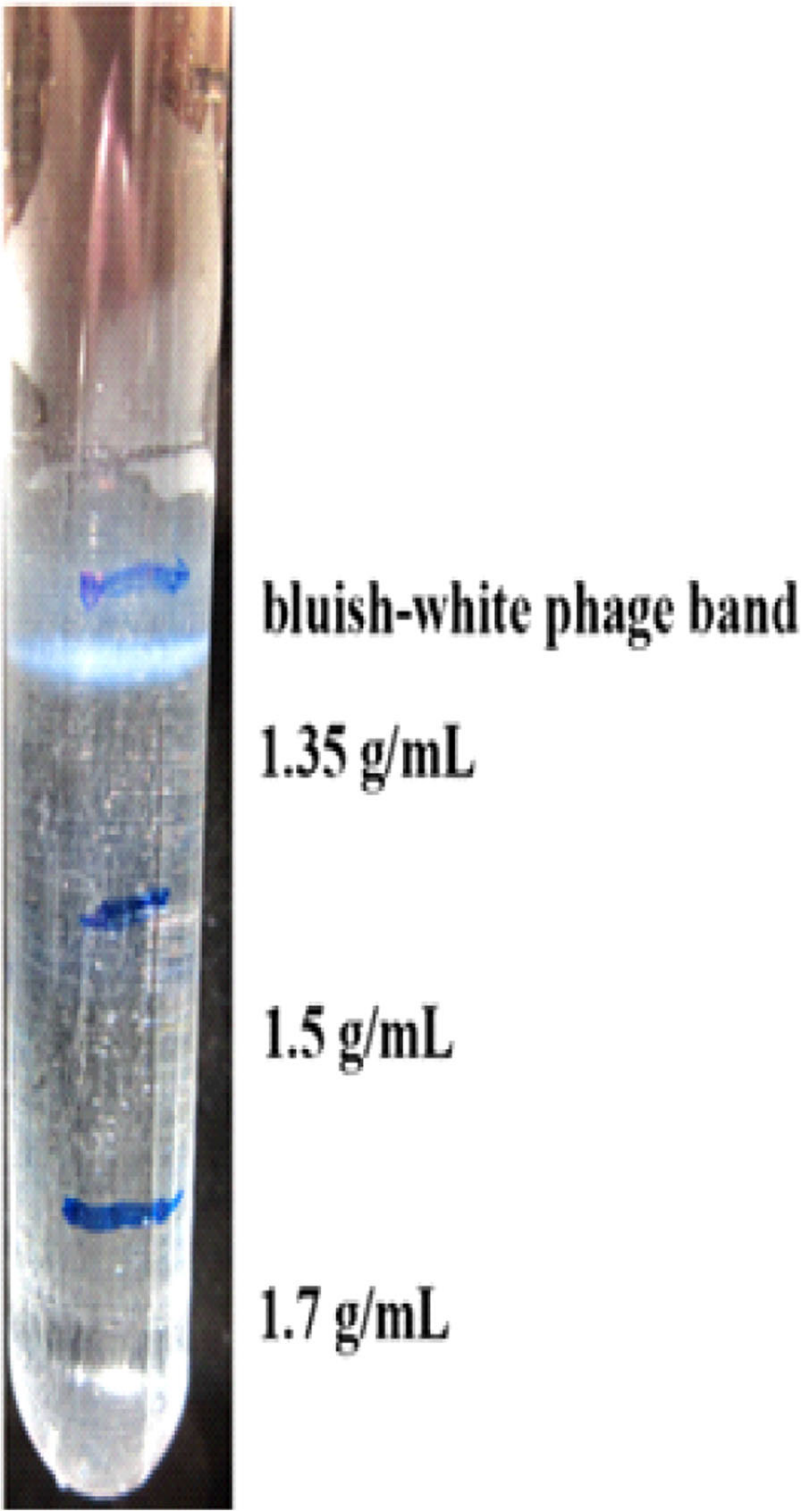
CsCl purification of the phages. The phages are in the bluish-white band

The phage particles were purified for electron microscopy via centrifugation in cesium chloride (CsCl) step gradients at different CsCl solution densities (1.35, 1.50, and 1.7 g/mL). The CsCl gradient solutions were prepared in a 36.5 mL Beckman polyallomer ultracentrifuge tubes using Beckman swinging bucket rotor (SW 40 Ti) (Beckman Coulter, California, USA). The final concentration of the CsCl solutions was estimated based on the formula as provided by [75]. Subsequently, a layer of the phage lysate was added (5 mL) and the volume was adjusted with SM buffer. Following ultracentrifugation at 35,000 rpm for 6 h at 4 °C, the observed bluish-white phage bands (Fig. 14) was collected and the CsCl was removed by dialysis using (10 K) MWCO slide-A-Lyzer dialysis cassettes as described by [76].

### Electron microscopic imaging of the phages

The phage suspensions were held on a Formvar carbon-coated copper grid which was glow-discharged by 0.01% poly-L-lysine for 5 min before being rinsed in sterilized dH_2_O. After rinsing, the suspensions were dried for 5 min to reduce the hydrophobicity level [77, 78]. A 5 μL aliquot of the phage suspensions (> 10^8^ PFU/mL) were transferred to the grid and negatively stained in the dark with 10 μL of 2% w/v uranyl acetate for 3 min. The excess stain was quickly removed by draining with the edge of a filter paper. The grids were air-dried for 2 min and their electro-micrographs were captured at magnifications between 100,000X and 300,000X using a HITACHI H-7100 TEM set at 100 kV.

### Phage DNA extraction

The genomic DNA of the isolated phages was extracted using gradient solutions of cesium chloride (CsCl). Sixty microliter of proteinase K (100 μg/mL) followed by 40 μL of 0.5 M EDTA pH 8 were added to each 1 mL volume of the dialyzed phage particles with a density of greater than 10^10^ PFU/mL. The mixture was vortexed for 10 s and incubated overnight at 37 °C. The extraction of the phage genome from the overnight culture was completed using a phage genome extraction kit (Norgen Biotek Corp, Canada) following the manufacturer’s instructions.

### DNA sequencing, assembly, annotation and bioinformatics analysis of the phage genome

Next generation sequencing was performed by using the Illumina HisSeq4000 platform, 2 × 150 bp paired-ends run. The sequence data was analyzed in three phases; in the first phase, the quality of the raw data was checked using FastQC v 0.10.1 before using FastX-toolkit v 0.0.13.2 to remove low quality values readings, ambiguous bases, and artifact reads. The readings were quality-trimmed or filtered to generate high quality readings used in the second phase which involves a whole genome de novo assembly. The Velvet assembler v1.2.10 was used for the de novo assembly of the clean reads with the following options were invoked: exp_cov auto, read_trkg yes, unused_reads yes, ins_length (UPMK_1 = 277; UPMK2 = 277), ins_length_sd (UPMK_1 = 23; UPMK2 = 27). The remaining parameters were left in default. Kmer length were optimised for both phage assembly as evident in the Additional files 1 and 2, where the best kmer for the assembly for both genomes was selected. For phage UPMK_1, its assembly was further improved by scaffolding and gapfilling using SSPACE v2.0 and Gapfiller v1.10, respectively. While for UPMK_2 only the dot plot graph of the assembled contig was visualised using Gepard v1.3. In phase three, genome annotation was carried out with Prodigal gene prediction pipeline (ver.2.6). As a follow-up to phase three, the predicted genes (> 33 aa) were annotated by protein BLAST search against the NCBI non-redundant database. Homology matches with a maximum e-value cut-off at 1.0 e^− 5^ were considered as significant hits. The tRNA and rRNA annotations were done using tRNAscan-SE (ver.1.3.1) and RNAmmer (ver.1.2), respectively. The phage genome was analyzed using the PHASTER (http://phast.wishartlab.com/) [79, 80]. Then, this analysis was invested to build the phylogenetic tree that was constructed with the maximum likelihood method by using Molecular Evolutionary Genetics Analysis version 7 (MEGE7) [81, 82]. Data were retrieved from GenBank (https://www.ncbi.nlm.nih.gov/ nucleotide). Further, Integrase nucleotide gene sequences obtained from whole phage genome from NCBI nucleotide database or from the published Staphylococcus genome were collected to determine the divergence of integrase gene in UPMK_1 and 2 and their clusters with the related phages as described in [83].

### Accession numbers

The genome sequences of phage UPMK_1 and UPMK_2 have been deposited in the GenBank under accession numbers MG543995 and MG564297, respectively.

### Assessment of biofilm-degradation in an in vitro and in situ static biofilm conditions

The 96-well polystyrene tissue culture microtiter plates were used for biofilm production assessment as previously described. Following several washing steps, phage MOI ~1 treated biofilms were assessed for biomass density after 2, 4, 6, 8, 12, and 24 h of treatment via staining with crystal violet and measuring the OD_570_ as described by Dakheel et al. [42]. The viable cells and phage particles within the treated biofilms and control were estimated. The biofilm content in each well was recovered by scraping, pipetting and collection into Eppendorf tubes. The tubes were centrifuged immediately, and the supernatants were serially diluted with SM buffer for phage particles count. For the viable cell counts, the pellets were resuspended in TSB and serially diluted. These tests were performed in triplicates.

Confocal laser scanning microscopy (CLSM) was performed using a modified cell culture slide for the in situ biofilm degradation assessment. The slide was prepared by substituting the bottom slide of the cell culture slide (4 wells) with a large glass cover slip. A specific piece of plastic was used to fix the cell well on the cover slip to prevent leaking during the culturing of the MRSA. These 4 wells were washed extensively (five times) with sterile dH_2_O and 70% ethanol, before being sterilized under UV light. To each well 10% of Poly-L- lysine was added and incubated overnight at 4 °C. The poly-L-lysine was removed and subsequently washed three times with sterile dH_2_O and dried before the seeding of the MRSA. The surface of the 48 h established biofilm on the well was carefully washed twice with normal saline (0.85% NaCl). The biofilm was then treated with the bacteriophage at MOI ~ 1 for a certain period depending on the best period to achieve removal by the phage in the microtiter plate. Thereafter, the supernatant was removed and the biofilm on the slide was washed twice, and stained with Filmtracer™ LIVE/DEAD Biofilm Viability Kit (Invitrogen Ltd., UK) following the manufacturer’s instructions. The stained biofilm was gently rinsed with normal saline thrice for a complete stain removal. Similarly, controls were prepared in the same manner but were rather treated with SM buffer. The biofilm images were obtained using a Leica CLSM model TCS SP5II (Leica Microsystems CMS GmbH, Germany). The biofilms were observed using the 40x objective lens (HCX PL APO lambda blue 40x/1.25 OIL UV) with a scan format of 1024 × 1024 resolution. Argon laser was used at 488 nm to excite SYTO9, and the fluorescent emission was detected at 495 to 535 nm. Propidium iodide (PI) was excited at 543 nm with a HeNe laser and its fluorescent emission was detected at 589 to 717 nm. Optical sections with z-step size 0.29 μm were obtained based on the thickness of the biofilms, and images were obtained randomly from selected positions of the wells. The resulting stack of images was analyzed and reconstructed in 3D using a Leica confocal software, (Lecia LASX version: 3.1.0.15537, Lecia Microsystems CMS GmbH, Germany).

### Statistical analysis

The statistical analysis of the obtained data was performed using SPSS Statistics 21 for windows (IBM) via the Student’s t test. The outcome of the data analysis was expressed as mean values ± standard deviation, and all the tests were performed in three independent replicates. Significance was considered at *P*-value less than 0.05.

## Additional files

Additional file 1: Optimization of assembly Kmer length and selection of the optimal assembly for UPMK_1. (PDF 88 kb)

Additional file 2: Optimization of assembly Kmer length and selection of the optimal assembly for UPMK_2. (PDF 95 kb)

Additional file 3: General features of putative ORFs from methicillin resistant *S.aureus* phage UPMK_1 with best matches in the NCBInr database. (PDF 258 kb)

Additional file 4: General features of putative ORFs from methicillin resistant *S.aureus* phage UPMK_2 with best matches in the NCBInr database. (PDF 159 kb)

Additional file 5: Dotplot of phage UPMK_2 contig assembly. (PDF 143 kb)
