# Supplementary material for: Genomic analyses of two novel biofilm-degrading methicillin-resistant Staphylococcus aureus phages
Source: BMC Microbiol. 2019 May 28;19:114. doi: 10.1186/s12866-019-1484-9 (PMC6540549; doi:10.1186/s12866-019-1484-9)
Supplement: Supplementary file 1 — Optimization of assembly Kmer length and selection of the optimal assembly for UPMK_1. (PDF 88 kb) [file 12866_2019_1484_MOESM1_ESM.pdf]

Additional file 1. Optimization of assembly Kmer length and selection of the optimal assembly for UPMK_1

Kmer	Contigs	N50	Total Assembly Size
65	6168	549	2320057
79	3535	736	1609634
85	969	9072	980819
95	384	41629	746114
99	247	136957	631281
105	2626	3541	988412
109	1065	19491	721869
111	896	19493	687286
113	765	16160	644637
119	369	17524	490791
125	258	14167	390211
129	251	5153	345973
133	173	3580	222113
135	87	5542	152788
137	32	11156	62112
139	1	40815	40815
141	1	40815	40815
