# Supplementary material for: Genomic analyses of two novel biofilm-degrading methicillin-resistant Staphylococcus aureus phages
Source: BMC Microbiol. 2019 May 28;19:114. doi: 10.1186/s12866-019-1484-9 (PMC6540549; doi:10.1186/s12866-019-1484-9)
Supplement: Supplementary file 2 — Optimization of assembly Kmer length and selection of the optimal assembly for UPMK_2. (PDF 95 kb) [file 12866_2019_1484_MOESM2_ESM.pdf]

Additional file 2. Optimization of assembly Kmer length and selection of the optimal assembly for UPMK_2

Kmer	Contigs	N50	Total Assembly Size
55	8054	575	2801105
57	7738	569	2710063
59	7158	577	2593733
61	6859	575	2494705
63	6780	566	2424883
65	5981	595	2286813
67	5764	592	2198576
69	5147	629	2077715
73	4479	670	1890153
77	3804	750	1713543
81	3073	941	1533658
85	957	5281	980483
89	833	5860	930279
91	754	5975	900680
95	401	20726	741878
97	368	26553	725002
99	246	35894	603926
101	3450	1476	1102433
103	2963	1823	1037748
105	2539	2335	980390
109	1813	3629	855030
111	909	11219	657560
115	651	9571	591247
119	536	5123	522785
123	410	3679	439403
129	259	2565	281636
135	57	6621	103328
137	3	39183	42448
139	1	40815	40815
141	1	40815	40815
143	0	0	0
145	0	0	0
