# Supplementary material for: Genomic analyses of two novel biofilm-degrading methicillin-resistant Staphylococcus aureus phages
Source: BMC Microbiol. 2019 May 28;19:114. doi: 10.1186/s12866-019-1484-9 (PMC6540549; doi:10.1186/s12866-019-1484-9)
Supplement: Supplementary file 3 — General features of putative ORFs from methicillin resistant S.aureus phage UPMK_1 with best matches in the NCBInr database. (PDF 258 kb) [file 12866_2019_1484_MOESM3_ESM.pdf]

Additional file 3. General features of putative ORFs from methicillin resistant *S. aureus* phage UPMK_1 with best matches in the NCBInr database

ORF	Position(nt) bp		Gene length	direction	Representative similarity to proteins in database	Identity %	E-value	Accession no.
	Start	Stop						
1	842	1408	567	forward	Hypothetical protein	100	4.00E-128	WP_001092763
2	1882	2580	699	reverse	Lantibiotic ABC transporter protein	100	2.00E-172	WP_000581552
3	2577	3338	762	reverse	Hypothetical protein	100	4.00E-162	BAF67983
4	3335	4027	693	reverse	Lantibiotic immunity protein F	100	0	WP_000691541
5	4050	5423	1374	reverse	Peptidase S8	53.5	4.00E-63	P30197
6	5433	5951	519	reverse	Epidermin decarboxylase	100	0	WP_000566596
7	5967	7211	1245	reverse	Lanthionine synthetase	100	0	WP_045176870
8	7204	10197	2994	reverse	Bacteriocin biosynthesis protein	59	9.00E-11	P21838
9	10262	10405	144	reverse	Lantibiotic gallidermin	100	3.00E-14	JQ655767
10	10819	11046	227	reverse	Hypothetical protein	99	0	Q6G8A9
11	11267	12250	984	reverse	Leucotoxin LukDv	100	0	Q2FXB0
12	12252	13172	920	reverse	Leucotoxin LukEv	-	-	Not available
13	14084	14182	99	forward	Hypothetical protein	100	0	WP_045176868
14	14552	15340	789	forward	Hypothetical protein	94.4	3.00E-14	ABX29824
15	16793	17137	344	forward	Hypothetical protein USA300HOU_1821	-	-	Not available
16	17134	17253	119	forward	Hypothetical protein	100	6.00E-17	YP_003857112
17	17247	17621	374	reverse	Hypothetical protein SAP26_gp44	100	1.00E-155	YP_008320216
18	17815	17973	158	forward	Hypothetical protein SA13_16	100	0	WP_000190226
19	17952	18722	771	forward	Hypothetical protein	100	0	WP_000803028
20	18732	19505	773	forward	DNA replication protein DnaC	100	3.00E-29	EUS87615
21	19499	19657	158	forward	Hypothetical protein O278_02737, partial	100	2.00E-44	WP_001123681
22	19671	19892	221	forward	Hypothetical protein	100	7.00E-92	WP_000049798
23	19996	20307	311	forward	Hypothetical protein	100	2.00E-34	WP_016028253
24	20312	20497	185	forward	Hypothetical protein	100	4.00E-82	WP_016028254

25	20498	20866	368	forward	Hypothetical protein	100	1.00E-51	WP_000131389
26	20870	21112	242	forward	Hypothetical protein	100	1.00E-50	BAB95284
27	21124	21378	254	forward	Hypothetical protein	100	5.00E-31	CRI18598
28	21368	21541	173	forward	Conserved hypothetical protein	100	6.00E-27	BAB95281
29	21824	21985	161	forward	Hypothetical protein	100	1.00E-125	WP_001061841
30	22000	22533	533	forward	Hypothetical protein	100	2.00E-48	WP_001282074
31	22579	22815	236	forward	Hypothetical protein	100	4.00E-36	WP_000195817
32	22812	23000	188	forward	Hypothetical protein	100	1.00E-37	NP_058494
33	22975	23175	200	forward	Unnamed protein product	95	3.00E-35	Q03183
34	23163	23351	188	forward	Transcriptional activator rinB	100	3.00E-92	WP_000286968
35	23352	23753	401	forward	Hypothetical protein	100	1.00E-63	WP_029550564
36	24103	24414	311	forward	Hypothetical protein	99	3.00E-112	WP_000594083
37	24831	24998	167	forward	Terminase	100	0	WP_031927907
38	24991	26199	1208	forward	Terminase	100	0	WP_001159672
39	26153	27631	1478	forward	Portal protein	99	0	ADL65886
40	27570	28550	980	forward	Phage minor head protein	100	2.00E-135	WP_043044228
41	28648	29238	590	forward	Capsid assembly scaffolding protein	100	0	WP_043044229
42	29254	30066	812	forward	Major capsid protein	100	7.00E-70	SBE97147
43	30083	30409	326	forward	Rho termination factor domain-containing protein	100	1.00E-69	YP_240825
44	30409	30723	314	forward	Head completion protein	100	1.00E-74	WP_000482986
45	30716	31051	335	forward	Phage head-tail adapter protein	99.3	3.00E-94	WP_001151332
46	31101	31451	350	forward	Hypothetical protein	100	5.00E-101	WP_015967254
47	31464	31901	437	forward	Tail completion protein	99.5	1.00E-132	A9CRB8
48	31888	32448	560	forward	Putative tail protein	99.4	2.00E-114	WP_000141084
49	32510	33004	494	forward	Hypothetical protein	100	2.00E-76	EYF55880
50	33025	33366	341	forward	Hypothetical protein	100	0	WP_031921309
51	33369	36338	2969	forward	Putative tape measure protein	100	0	WP_031897669
52	36680	37288	608	forward	Phage tail protein	99.8	0	WP_051121127
53	37299	39185	1886	forward	Peptidase	99.7	0	WP_042908142
54	39198	41096	1898	forward	Hypothetical protein	-	-	Not available

55	41700	42680	980	forward	Hypothetical protein	100	0	P0A0B3
56	42682	44274	1592	forward	Phosphoenolpyruvate carboxykinase	100	0	WP_000345609
57	44527	45273	746	reverse	Dipeptidyl aminopeptidase	100	3.00E-127	EES95489
58	45278	45811	533	reverse	Nucleoside triphosphatase YtkD	100	3.00E-57	P67305
59	45817	46074	257	forward	Hypothetical protein	100	0	WP_045177246
60	46071	47072	1001	reverse	O-succinylbenzoate synthase	97.5	0	P63526
61	47077	48555	1478	reverse	2-succinylbenzoate-CoA ligase	100	8.00E-114	WP_045177252
62	48714	49154	404	reverse	Hypothetical protein	100	4.00E-110	WP_049296628
63	49472	50122	650	forward	Calcium-binding protein	100	0	WP_049296627
64	50203	51198	995	forward	Hypothetical protein (DUF4352 domain-containing protein)	100	7.00E-150	WP_045177256
65	51274	51900	626	forward	Hypothetical protein	100	1.00E-75	WP_045177258
66	51941	52282	341	forward	Hypothetical protein	100	3.00E-136	WP_045177260
67	52779	52955	176	forward	Hypothetical protein	100	3.00E-127	WP_045177261
68	53153	53626	473	reverse	Hypothetical protein	100	1.00E-169	Q2FXC2
69	53992	54699	707	forward	Serine protease SplA	100	3.00E-171	Q6G8C2
70	54782	55546	764	forward	Serine protease SplB	100	3.00E-170	Q8NVX7
71	55604	56323	719	forward	Serine protease SplC	99.5	2.00E-171	Q7A2Q9
72	56444	57163	719	forward	Serine protease SplD	100	3.00E-168	Q5HEW4
73	57321	58037	716	forward	Serine protease SplE	100	8.00E-172	Q5HEW5
74	58188	58907	719	forward	Serine protease SplF	100	0	WP_000028669
75	59270	60826	1556	forward	Type I restriction-modification system subunit M	100	0	WP_000072624
76	60819	62018	1199	forward	Specificity determinant HsdS	100	8.00E-120	KIX70484
77	63214	63732	518	forward	Tail protein, partial	-	-	Not available
78	63738	63968	230	forward	Hypothetical protein	100	2.00E-86	WP_015978410
79	63958	64353	395	forward	Hypothetical protein	100	1.00E-99	EUK61875
80	64409	64846	437	forward	Phage phi LC3 family holin, partial	90.5	0	P24556
81	64812	66272	1460	forward	Phage lysin	100	3.00E-67	WP_000344116
82	66669	66980	311	forward	Hypothetical protein	100	1.00E-85	WP_001251275
83	66967	67350	383	forward	Hypothetical protein	42.5	2.00E-18	P03015

84	67497	68882	1385	reverse	Serine recombinase gin	100	3.00E-160	WP_000392109
85	69089	69769	680	reverse	Hypothetical protein	100	2.00E-171	WP_000358224
86	69805	70524	719	reverse	Transcriptional regulator	100	2.00E-44	WP_001198672
87	70666	70884	218	forward	Transcriptional regulator	100	3.00E-180	WP_001573849
88	70900	71637	737	forward	Repressor protein	100	5.00E-41	WP_000455728
89	71650	71859	209	forward	Hypothetical protein	100	2.00E-27	WP_000048126
90	71873	72034	161	forward	Hypothetical protein	100	2.00E-52	WP_000291487
91	72127	72387	260	forward	Hypothetical protein	100	1.00E-44	WP_000815400
92	72397	72618	221	forward	Hypothetical protein	100	2.00E-151	WP_000139720
93	72611	73234	623	forward	Hypothetical protein	90.7	1.00E-72	Q932A8
94	73234	73659	425	forward	Single-stranded DNA-binding protein	100	4.00E-131	WP_001004506
95	73670	74221	551	forward	DNA endonuclease I-HmuI	98.6	2.00E-165	YP_240792
96	74222	74896	674	forward	Hypothetical protein	100	2.00E-78	Q2FXE6
97	75356	75721	365	forward	Putative fluoride ion transporter CrcB 1	99	4.00E-75	Q2FXE5
98	75718	76071	353	forward	Putative fluoride ion transporter CrcB 2	56.5	3.00E-97	O32210
99	76454	77287	833	reverse	Glyoxal reductase	100	0	BAB42875
100	77499	78407	908	reverse	Hypothetical protein	99.7	0	Q8NVZ9
101	78532	79725	1193	reverse	S-adenosylmethionine synthase	100	9.00E-68	WP_000836465
102	80025	80336	311	reverse	Rhodanese-like domain-containing protein	99.8	0	Q8NW17
103	80358	82667	2309	reverse	Leucine-tRNA ligase	54.9	9.00E-134	O34546
104	83069	84250	1181	reverse	Putative MFS-type transporter YttB	100	0	WP_045177230
105	84632	85663	1031	forward	Riboflavin biosynthesis protein RibD	100	6.00E-148	WP_000493888
106	85670	86302	632	forward	Riboflavin synthase subunit alpha	100	0	Q6G8G1
107	86313	87494	1181	forward	Riboflavin biosynthesis protein RibBA	100	1.00E-107	P61595
108	87507	87971	464	forward	6,7-dimethyl-8-ribityllumazine synthase	100	0	WP_001196345
109	88093	89094	1001	reverse	Proline dehydrogenase	95.7	1.00E-53	SCU55093
110	90818	91102	284	reverse	Hypothetical protein	-	-	Not available
111	91261	93582	2321	forward	Hypothetical protein	-	-	Not available
112	93692	93853	161	forward	Hypothetical protein	100	5.00E-57	EPX93583
113	93909	94184	275	forward	Holin, partial	99.7	0	WP_001141518

114	94171	95583	1412	forward	Amidase	100	2.00E-124	WP_001035623
115	95644	96201	557	reverse	Hypothetical protein	100	7.00E-40	WP_000125170
116	96862	97068	206	forward	Hypothetical protein	100	9.00E-40	WP_000498229
117	97082	97282	200	forward	Hypothetical protein	74	9.00E-53	P30338
118	98128	98442	314	forward	Arsenical resistance operon repressor	99.7	0	Q8NW09
119	98442	99734	1292	forward	Arsenical pump m	100	6.00E-94	Q5HF01
120	99752	100147	395	forward	Protein ArsC	100	1.00E-40	EEW44025
121	101346	101555	209	reverse	Truncated transposase	100	1.00E-40	EEW44025
122	101758	101901	143	reverse	Transposase	100	4.00E-25	WP_001549059
123	102832	104844	2012	reverse	Hypothetical protein	-	-	Not available
124	105968	106234	266	reverse	Hypothetical protein	100	2.00E-56	WP_050003995
125	106701	106916	215	forward	Hypothetical protein	-	-	Not available
126	106942	108333	1391	forward	Acetyl-coenzyme A synthetase	73.2	0	P39062
127	108743	109264	521	forward	RNA polymerase sigma factor SigS	100	3.00E-81	Q6G8F3
128	109807	110250	443	reverse	Hypothetical protein	100	3.00E-97	WP_001030463
129	110678	111115	437	forward	TIGR01212 family radical SAM protein	98.9	6.00E-140	KAG35383
130	111112	111675	563	forward	Putative rRNA methylase YtqB	52	4.00E-58	O34614
131	112018	112245	227	reverse	Hypothetical protein	-	-	Not available
132	112574	113197	623	reverse	Hypothetical protein	-	-	Not available
133	113471	113761	290	forward	Phage terminase small subunit	100	3.00E-62	SCT09172
134	113864	114745	881	forward	Hypothetical protein	-	-	Not available
135	114682	115941	1259	reverse	Hypothetical protein	-	-	Not available
136	116429	116602	173	reverse	Uncharacterised protein	100	1.00E-31	SCU35234
137	116908	117264	356	forward	Hypothetical protein	-	-	Not available
138	117241	118020	779	reverse	Antirepressor	100	0	WP_031916499
139	118020	118136	116	reverse	Hypothetical protein	-	-	Not available
140	123353	123652	299	reverse	Hypothetical protein	98.9	1.00E-61	WP_061737271
141	123652	123879	227	reverse	Hypothetical protein	-	-	Not available
142	123919	124092	173	reverse	Hypothetical protein	100	2.00E-31	WP_000977107
143	124096	124473	377	reverse	Hypothetical protein	100	3.00E-83	WP_000705896

144	124759	125409	650	reverse	D-3-phosphoglycerate dehydrogenase, partial	99.6	3.00E-170	KMR60721
145	125641	126225	584	forward	Tail fiber protein	99.5	9.00E-136	CUG85000
146	126330	126536	206	forward	Hypothetical protein	-	-	Not available
147	126644	126826	182	reverse	Hypothetical protein	100	3.00E-34	WP_000411343
148	126827	127204	377	reverse	Hypothetical protein	100	1.00E-83	WP_000705919
149	134936	135151	215	forward	Pyridine nucleotide-disulfide oxidoreductase family protein	100	1.00E-92	CYB54786
150	136685	137017	332	forward	NAD(FAD)-utilizing dehydrogenase	100	2.00E-73	SCU54614
151	137253	137672	419	reverse	Hypothetical protein	-	-	Not available
152	138255	138404	149	reverse	Hypothetical protein	-	-	Not available
153	138692	138988	296	reverse	Hypothetical protein	100	5.00E-61	WP_029051944
154	144457	144588	131	forward	Hypothetical protein	-	-	Not available
155	146216	146428	212	reverse	Hypothetical protein	-	-	Not available
