# Supplementary material for: Genomic analyses of two novel biofilm-degrading methicillin-resistant Staphylococcus aureus phages
Source: BMC Microbiol. 2019 May 28;19:114. doi: 10.1186/s12866-019-1484-9 (PMC6540549; doi:10.1186/s12866-019-1484-9)
Supplement: Supplementary file 4 — General features of putative ORFs from methicillin resistant S.aureus phage UPMK_2 with best matches in the NCBInr database. (PDF 159 kb) [file 12866_2019_1484_MOESM4_ESM.pdf]

Additional file 4. General features of putative ORFs from methicillin resistant *S. aureus* phage UPMK_2 with best matches in the NCBInr database

ORF	Position(nt) bp		Gene length	Direction	Representative similarity to proteins in database	Identity %	E-value	Accession no.
	Start	Stop						
1	50	169	120	forward	Hypothetical protein	100	6.00E-17	YP_003857112
2	163	813	651	reverse	Hypothetical protein	100	1.00E-155	YP_008320216
3	868	1638	771	forward	Hypothetical protein	100	0	WP_000190226
4	1648	2421	774	forward	DNA replication protein DnaC	100	0	WP_000803028
5	2415	2573	159	forward	Hypothetical protein	100	3.00E-29	EUS87615
6	2587	2808	222	forward	Hypothetical protein	100	2.00E-44	WP_001123681
7	2819	3223	405	forward	Hypothetical protein	100	7.00E-92	WP_000049798
8	3228	3413	186	forward	Hypothetical protein	100	2.00E-34	WP_016028253
9	3414	3782	369	forward	Hypothetical protein	100	4.00E-82	WP_016028254
10	3786	4028	243	forward	Hypothetical protein	100	1.00E-51	WP_000131389
11	4040	4294	255	forward	Hypothetical protein	100	2.00E-51	BAB95284
12	4284	4457	174	forward	Conserved hypothetical protein	100	5.00E-31	CRI18598
13	4458	4739	282	forward	Hypothetical protein	100	7.00E-59	WP_000454993
14	4740	4901	162	forward	Hypothetical protein	100	6.00E-27	BAB95281
15	4916	5449	534	forward	Dimeric dUTPase	99.4	1.00E-125	WP_061839007
16	5495	5731	236	forward	Hypothetical protein	100	4.00E-36	WP_000195817
17	5728	5916	188	forward	Hypothetical protein	100	1.00E-37	NP_058494
18	5891	6091	200	forward	Hypothetical protein	-	-	Not available
19	6079	6267	188	forward	Phage transcriptional activator rinb	93	4.00E-31	Q03183
20	6268	6669	402	forward	Hypothetical protein	100	3.00E-92	WP_000286968
21	7019	7330	312	forward	Hypothetical protein	100	1.00E-63	WP_029550564
22	7420	7914	495	forward	Phage terminase small subunit	99.3	3.00E-112	WP_000594083
23	7907	9115	1209	forward	Phage terminase large subunit	100	0	WP_031927907
24	9069	10547	1419	forward	Phage portal protein	100	0	EIK05322
25	10486	11466	980	forward	Phage minor head protein	99	0	ADL65886
26	11564	12154	591	forward	Capsid assembly scaffolding protein	98.4	3.00E-133	SBF22489

27	12170	12982	813	forward	Putative phage major capsid protein	98.8	0	SBF22506
28	12999	13325	327	forward	Phage transcriptional terminator (factor Rho domain- containing protein)	100	7.00E-70	SBE97147
29	13325	13639	315	forward	Head completion protein gp15	100	1.00E-69	YP_240825
30	13632	13967	336	forward	Putative head-tail adaptor	100	1.00E-74	WP_000482986
31	13954	14367	414	forward	Hypothetical protein	99.2	3.00E-94	WP_001151332
32	14380	14817	438	forward	Tail completion protein gp17	100	5.00E-101	WP_015967254
33	14804	15364	561	forward	Putative tail protein	99.4	2.00E-130	WP_000046067
34	15426	15920	495	forward	Hypothetical protein	99.3	2.00E-114	WP_000141084
35	15941	16282	342	forward	Hypothetical protein	100	2.00E-76	EYF55880
36	16285	19254	2970	forward	Tap measure protein	100	0	WP_031921309
37	19269	20204	936	forward	Phage tail protein	100	0	WP_031897669
38	20215	22101	1887	forward	Peptidase	100	0	WP_051121127
39	22114	24012	1899	forward	Putative minor structural protein	99.6	0	WP_033859169
40	24012	25835	1824	forward	Hypothetical protein	99.6	0	WP_000259636
41	25835	26212	378	forward	Hypothetical protein	100	3.00E-83	WP_000705896
42	26216	26389	174	forward	Hypothetical protein	100	2.00E-31	WP_000977107
43	26429	26728	336	forward	Hypothetical protein	99	2.00E-70	EFT84252
44	26865	28739	1875	forward	Mannosyl-glycoprotein endo-beta-N-acetylglucosaminidase	99.6	0	WP_023487054
45	28752	29924	1173	forward	Tail fiber protein	100	0	WP_000276656
46	29930	30325	396	forward	Hypothetical protein	100	2.00E-86	WP_015978410
47	30381	30818	438	forward	Phage phi LC3 family holin	100	1.00E-99	EUK61875
48	30799	32244	1446	forward	Phage lysin, N-acetylmuramoyl-L-alanine amidase.	90.5	0	P24556
49	32641	32952	312	forward	Hypothetical protein	99	5.00E-67	WP_060474088
50	32939	33322	384	forward	Hypothetical protein	99	2.00E-84	KLM17145
51	33469	34854	1386	reverse	Serine recombinase gin	99.7	0	WP_000861313
52	35061	35741	681	reverse	Phage repressor	99.5	8.00E-160	WP_031925815
53	35777	36496	720	reverse	Peptidase S24	100	9.00E-171	WP_064262879
54	36638	36856	219	forward	Cro-like repressor	100	7.00E-44	WP_001198673

55	36872	37597	726	forward	Phage repressor protein	100	2.00E-177	WP_001573849
56	37622	37831	209	forward	Hypothetical protein	100	5.00E-41	WP_000455728
57	38099	38359	260	forward	Hypothetical protein	98.8	8.00E-52	WP_061644690
58	38369	38590	222	forward	Hypothetical protein	100	1.00E-44	WP_000815400
59	38583	39206	624s	forward	Hypothetical protein	100	2.00E-151	WP_000139720
60	39206	39631	426	forward	Single-stranded DNA-binding	99	2.00E-96	WP_052995597
61	39642	40193	552	forward	DNA endonuclease I-HmuI	99.4	8.00E-131	WP_031797824
62	40194	40868	675	forward	Hypothetical protein	99	9.00E-166	YP_240792
