# Supplementary material for: Genomic analyses of two novel biofilm-degrading methicillin-resistant Staphylococcus aureus phages
Source: BMC Microbiol. 2019 May 28;19:114. doi: 10.1186/s12866-019-1484-9 (PMC6540549; doi:10.1186/s12866-019-1484-9)

Additional file 5. Dotplot of phage UPMK_2 contig assembly. Corner showed overlap length: 140bp with percentage mismatch: 0.0, presence of overlaps is a characteristic of circular DNA

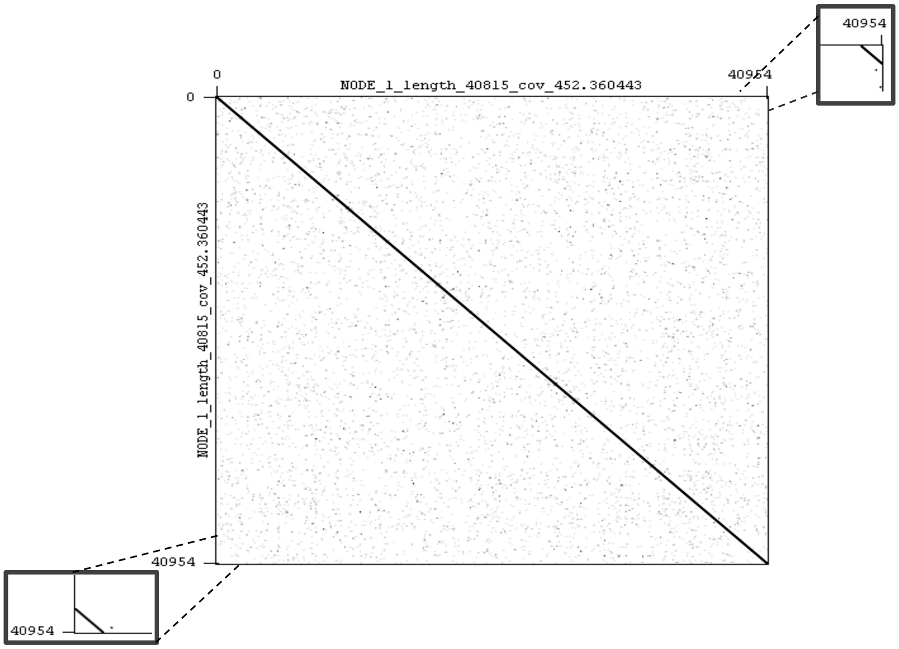

Supplement: Supplementary file 5 — Dotplot of phage UPMK_2 contig assembly. (PDF 143 kb) [file 12866_2019_1484_MOESM5_ESM.pdf]
